# G-quadruplex-dependent transcriptional regulation by molecular condensation in the *Bcl3* promoter

**DOI:** 10.1093/nar/gkaf827

**Published:** 2025-08-30

**Authors:** Wanki Yoo, Yi Wei Song, Varun Bansal, Kyeong Kyu Kim

**Affiliations:** Department of Precision Medicine, Graduate School of Basic Medical Science (GSBMS), Institute for Antimicrobial Resistance Research and Therapeutics, Sungkyunkwan University School of Medicine, Suwon 16419, Republic of Korea; Department of Precision Medicine, Graduate School of Basic Medical Science (GSBMS), Institute for Antimicrobial Resistance Research and Therapeutics, Sungkyunkwan University School of Medicine, Suwon 16419, Republic of Korea; Department of Precision Medicine, Graduate School of Basic Medical Science (GSBMS), Institute for Antimicrobial Resistance Research and Therapeutics, Sungkyunkwan University School of Medicine, Suwon 16419, Republic of Korea; Department of Precision Medicine, Graduate School of Basic Medical Science (GSBMS), Institute for Antimicrobial Resistance Research and Therapeutics, Sungkyunkwan University School of Medicine, Suwon 16419, Republic of Korea

## Abstract

G-quadruplexes (G4s) are pivotal in transcriptional regulation. Although the interaction between G4s and G4-binding transcription factors (TFs) is critical for G4-dependent transcriptional regulation, the detailed mechanism, especially TF enrichment at G4s and its correlation with transcriptional regulation, remains unknown. In this study, using specificity protein 1 (SP1) as a representative G4-binding TF, we examined the mechanism of G4-dependent transcriptional regulation. Genomic analysis revealed substantial enrichment of SP1 in the oncogenic *Bcl3* promoter harboring G4-forming sequences. We demonstrated that the formation of transcriptional condensates and the transcriptional activation of the *Bcl3* promoter are heavily dependent on G4-dependent SP1 binding. Moreover, dissociation of SP1 condensates was prompted by RNA, which was enhanced by G4 formation within the RNA. Collectively, these results underscore the pivotal role of G4 in regulating gene expression through the modulation of SP1-mediated transcriptional condensation.

## Graphical Abstract

**Graphical Abstract:**
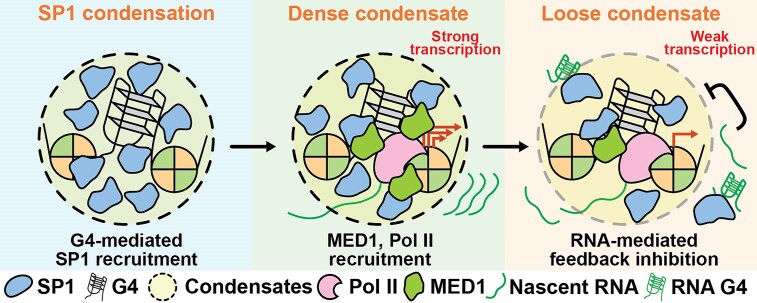

## Introduction

DNA and RNA can be folded into various types of non-canonical structures, including a hairpin, Z-DNA, triplexes, G-quadruplexes (G4s), i-motifs, and AC-motif [1–3]. These non-canonical DNA and RNA structures play crucial roles in genome integrity, epigenetic regulation of chromatin, and gene regulation [4, 5]. Among them, G4s, four-stranded structures formed by successive guanine (G) quartets bonded via Hoogsteen hydrogen bonds, are well-known for their roles in regulating both transcription and translation [6]. As G4s in promoters and 5′-UTRs are also known to regulate the expression of key oncogenes such as *MYC, BCL2, VEGF, c-KIT, TEAD4, and PDGFRB*, elucidating the role of G4 in gene expression regulation in cancers is of great importance [7–9].

In the current transcriptional activation model, liquid-like condensates formed by transcription factors (TFs) play a pivotal role in transcriptional activation by recruiting other TFs, coactivators, and transcriptional machinery into the transcriptional condensates (TCs) [10–12]. TFs become locally concentrated through interactions with DNA motif [13–15], making TC formation largely dependent on the binding affinity of TFs to these motifs, the number of motifs present, epigenetic modifications, and the local chromatin structure [13, 14, 16]. Furthermore, a study has shown that the presence of G4 structures in the *CCND1* promoter recruits *myc*-associated zinc-finger protein (MAZ) to the promoter, leading to TC formation and subsequent *CCND1* expression activation [17]. These findings highlight the importance of G4s in the genome, particularly in facilitating the condensation of G4-binding TFs. However, despite these observations, both the detailed mechanisms by which G4 structures dynamically regulate TC formation involving G4-binding TFs and transcription, and the therapeutic potential of targeting G4-associated TC formation, remain largely unexplored.

Among G4-binding TFs, specificity protein 1 (SP1) is known to regulate the expression of various genes, including *CCND1*, *MMP2*, and *HIF1A* [18–20]. SP1 is also directly involved in the regulation of many oncogenes, such as *IGF1R*, *hTERT*, *TP53*, and *CDKN1A*, by binding to G4s in their gene promoters [21–24]. Notably, SP1 plays a crucial role in promoting proliferation in breast cancer cells by regulating the expression of insulin-like growth factor-I receptor (IGF1R) [25]. Additionally, SP1 upregulation is consistently observed across various breast cancer subtypes, including hormone receptor-positive, HER2-positive, and triple-negative breast cancer [26–28]. Consequently, targeting SP1 has emerged as a potential therapeutic strategy for breast cancer treatment [29, 30]. At the molecular level, SP1 distinguishes itself from other SP family TFs through its ability to self-associate in a DNA-dependent manner via its *C*-terminal multimerization domain [31–33]. This feature is particularly important for TF condensation and TC formation [14, 15, 34]. A recent study further demonstrated that SP1 undergoes phase separation at super-enhancer regions [35]. Considering SP1’s unique attributes, including its role in regulating oncogene expression, association with G4 structures, and potential to facilitate TC formation, we selected SP1 as a model TF to investigate the role of G4s in TC formation.

To study the role of G4s in SP1 condensation at promoters and their implications for gene regulation, SP1-enriched promoters and their G4-forming potential (QGRS scores) were first analyzed using publicly available SP1 chromatin immunoprecipitation (ChIP)-seq data. Based on this analysis, the *bcl3* promoter was selected as a model SP1-enriched promoter, as it exhibited a higher QGRS score and the *bcl3* gene is strongly associated with breast cancer [36]. *In vitro* assays and a cell-based reporter assay demonstrated that G4 structure formation in the *Bcl3* promoter is necessary for SP1 binding and significantly enhances promoter activity by facilitating condensate formation. Co-localization of MED1 and RNA Pol II with SP1 condensates was also observed, indicating the formation of TCs involving SP1. Furthermore, RNA derived from the *Bcl3* transcript was found to dissolve SP1 condensates and inhibit transcription, which was enhanced by G4 formation within the RNA, suggesting the existence of a negative feedback mechanism mediated by RNA G4 structures. Collectively, our results demonstrate that G4s can regulate transcription by promoting TC formation through recruitment of SP1 to the promoter, followed by G4 RNA-mediated dissolution of these complexes.

## Materials and methods

### Genomic analysis

Publicly available SP1 ChIP-seq data for MCF-7 cells were downloaded from the Gene Expression Omnibus (GEO) database (GSM2423902). SP1 ChIP-seq data for A549, GM12878, and HCT116 cells were obtained using the Integrative Genomics Viewer (IGV) [37]. Additionally, MED1 ChIP-seq data for MCF-7 (GSM7027284), A549 (GSM2040033), GM12878 (GSM2443457), and HCT116 (GSM4490345) cells, as well as RNA Pol II ChIP-seq and RNA-seq data for MCF-7 cells (GSM822295 and GSM958745), were downloaded from the GEO database. ChIP-seq data for MAZ (GSM2423262), NRF1 (GSE91522), and E2F4 (GSE105536) in MCF-7 cells were also downloaded from the GEO database. All other ChIP-seq and RNA-seq data were accessed directly through the IGV software. ChIP-seq images were generated using IGV, and data analysis of ChIP-seq files was conducted using the Subio platform software (Subio Inc., Kagoshima, Japan).

### Circular dichroism spectroscopy, UV absorption melting curve analysis, and dimethylsulphate (DMS) footprinting

For G4 formation, wild-type (WT) and mutant (MT) oligos from the *Bcl3* promoter (chr19:45 251 889–45 251 918; 30 nt) were dissolved in G4 buffer containing 10 mM Tris-HCl (pH 7.5) and 100 mM KCl, heated at 95°C for 5 min, and then allowed to cool to 25°C for 2 h. To assess the effects of different salts on G4 formation, 100 mM NaCl or LiCl was used in place of KCl. To evaluate the effects of G4-binding ligands, TMPyP4 and pyridostatin (PDS) were co-incubated with oligos at a ligand:G4 molar ratio of 2:1. Circular dichroism (CD) spectra were recorded at 25°C between 220–320 nm, using a scanning speed of 200 nm/min, a response time of 1 s, a pitch of 1 nm, and a bandwidth of 2 nm. To determine melting temperatures, CD signals at 264 or 284 nm were monitored over a temperature range of 25–95°C. For UV absorption-based melting curve analysis, oligos were annealed as described above, and UV absorbance was monitored at 295 nm while the temperature increased from 25°C to 95°C. Both CD and UV measurements were performed using a Jasco J-810 spectrometer equipped with a Peltier temperature controller (JASCO, Tokyo, Japan). Dimethyl sulfate (DMS) footprinting experiments were conducted using 5′ FAM-labeled oligos. Additional nucleotides were added to minimize steric hindrance from the FAM label during G4 structure formation. G4s were formed as described above and treated with 0.5% DMS for 2 min. The reaction was quenched by adding 1 μg of calf thymus DNA, which was then separated on a 20% native polyacrylamide gel, extracted, and ethanol-precipitated. Samples were subsequently cleaved with 1 M piperidine at 95°C, dried, resuspended in alkaline sequencing dye, and separated on a 20% denaturing polyacrylamide gel.

### Thioflavin T and *N*-methyl mesoporphyrin IX fluorescence assay

For the Thioflavin T (ThT) fluorescence assay, G4 structures of *Bcl3* promoter WT and MT oligos were formed as described above. For the N-methyl mesoporphyrin IX (NMM) fluorescence assay, sense and antisense strands covering a 90-nucleotide region surrounding the *Bcl3* promoter G4-forming sequence (chr19:45 251 859–45 251 948; 90 nt) were annealed together in the presence or absence of 40% PEG200 or 10% PEG8000 to generate double-stranded DNA G4 structures with or without 100 mM KCl (dsBcl3-G4) [38, 39]. After annealing, 0.5 μM ThT and 1 μM NMM were mixed with 1 μM ssBcl3-G4 and 2 μM dsBcl3-G4, respectively. Following 1 h of incubation at 25°C, ThT and NMM fluorescence signals were measured by excitation at 415 and 393 nm, respectively. All experiments were performed in triplicate. Fluorescence measurements were conducted at 25°C using a Jasco FP-750 spectrofluorometer (JASCO, Tokyo, Japan).

### Atomic forced microscopy (AFM) analysis and CD analysis of dsBcl3 G4

For AFM analysis, dsBcl3-G4 or non-G4 samples were annealed at a final concentration of 10μM in the presence of 10% PEG8000, with either 1 mM or 100 mM KCl. Samples were diluted to 0.5 nM in the same buffer used for annealing, and 5 μL were deposited onto freshly cleaved mica in 10 mM HEPES-NaOH (pH 7.3), 25 mM KCl, and 10 mM MgCl_2_. After 5 min of incubation, the mica was rinsed with distilled water and imaged in air under intermittent tapping mode using a JPK Nanowizard ULTRA Speed (Bruker, Billerica, MA, USA). For CD analysis of dsBcl3-G4, samples were prepared as described above, except using different buffer conditions. CD spectra of 10 μM dsBcl3-G4 were recorded at 25°C between 220 and 320 nm, with a scanning speed of 200 nm/min, a response time of 1 s, a pitch of 1 nm, and a bandwidth of 2 nm. Buffer signals were subtracted. All CD measurements were performed using a Jasco J-810 spectrometer (JASCO, Tokyo, Japan).

### Bio-layer interferometric analysis

For bio-layer interferometric (BLI) analysis, 1 μM WT or MT pre-formed, biotin-labeled, single-stranded *Bcl3* promoter G4s were immobilized on a streptavidin chip (Sartorius, Göttingen, Germany), and the baseline was acquired using phosphate-buffered saline (PBS). Various concentrations of SP1 (Active Motif, Carlsbad, CA, USA) ranging from 0 to 6 μM were applied for association and dissociation in PBS. The equilibrium dissociation constant (*K*_D_), association rate constant (*k*_a_), and dissociation rate constant (*k*_d_) were calculated by global fitting using a 1:1 binding model in BLItz Pro v1.2.1.5 software (Sartorius, Göttingen, Germany). All BLI experiments were performed in PBS at room temperature. To determine *k*_a_ and *k*_d_, *k_obs_* was calculated as follows:


\begin{eqnarray*}
Y = {{Y}_0} + A\left( {1 - {{e}^{ - {{k}_{obs}}*t}}} \right)
\end{eqnarray*}

where Y is the level of binding (wavelength shift), Y_0_ is the binding at the start of association, A is an asymptote, and t is time. *k*_obs_ is the overall rate of combined association and dissociation of the two binding partners. The *k*_d_ was calculated using the following equation:


\begin{eqnarray*}
Y = Y0 + A{{e}^{ - {{k}_d}*t}}
\end{eqnarray*}

where Y_0_ is the binding at the start of the dissociation. The following equation was used to calculate *k*_a_:


\begin{eqnarray*}
{{k}_a} = \frac{{{{k}_{obs}} - {{k}_d}}}{{\left[ {Analyte} \right]}}
\end{eqnarray*}

Finally, *K*_D_, the affinity constant or equilibrium dissociation constant, was calculated using the following equation:


\begin{eqnarray*}
{{K}_D} = \frac{{{{k}_d}}}{{{{k}_a}}}
\end{eqnarray*}

For the two-step binding model, we assume sequential SP1 binding to G4 as follows:


\begin{eqnarray*}
G4 + SP1\ \leftrightarrow G4SP1 + SP1 \leftrightarrow G4{{\left( {SP1} \right)}_2}
\end{eqnarray*}

Using ordinary differential equations, the two-step sequential ligand binding can be transformed as follows [68]:


\begin{eqnarray*}
\frac{{d\left[ {G4} \right]}}{{dt}} = \ - {{k}_{on}}1 \cdot \left[ {G4} \right] \cdot \left[ {SP1} \right] + {{k}_{off}}1 \cdot \left[ {G4SP1} \right]
\end{eqnarray*}

\begin{eqnarray*}
&& \frac{{d\left[ {G4SP1} \right]}}{{dt}} =\nonumber\\ && \ {{k}_{on}}1 \cdot \left[ {G4} \right] \cdot \left[ {SP1} \right] - {{k}_{off}}1 \cdot \left[ {G4SP1} \right] - {{k}_{on}}2 \cdot \left[ {G4} \right] \cdot \left[ {SP1} \right] \nonumber\\ && + {{k}_{off}}2 \cdot \left[ {G4{{{\left( {SP1} \right)}}_2}} \right]
\end{eqnarray*}

\begin{eqnarray*}
&&\frac{{d\left[ {G4{{{\left( {SP1} \right)}}_2}} \right]}}{{dt}} =\nonumber\\ && \ {{k}_{on}}2 \cdot \left[ {G4SP1} \right] \cdot \left[ {SP1} \right] - {{k}_{off}}2 \cdot \left[ {G4{{{\left( {SP1} \right)}}_2}} \right]
\end{eqnarray*}

The total response is as follows:


\begin{eqnarray*}
R\left( t \right) = \ R1 \cdot \left[ {G4SP1} \right]\left( t \right) + R2 \cdot \left[ {G4{{{\left( {SP1} \right)}}_2}} \right]\left( t \right)
\end{eqnarray*}

where *k*_on_1 and *k*_off_1 are the association and dissociation rates of the first binding step, *k*_on_2 and *k*_off_2 are the association and dissociation rate of the second step in the two-step binding model. R1 and R2 are the maximum equilibrium binding responses of the first and second steps, respectively.

### Electrophoretic mobility shift assay

SP1 binding to DNA or RNA oligos was monitored using electrophoretic mobility shift assay (EMSA). Binding reactions were carried out in a total volume of 5 μL in binding buffer containing 10 mM HEPES-NaOH (pH 7.3), 50 mM KCl, 5 mM MgCl_2_, 0.5 mM DTT, 5% glycerol, and 0.5 mg/mL BSA. After 1 h of incubation, samples were resolved on a 5% non-denaturing polyacrylamide gel in 0.5 × TBE buffer at 70 V for 60 min at 4 °C and visualized by staining with SYBR Gold dye (Thermo Fisher Scientific, Waltham, MA, USA). Images were acquired using a ChemiDoc XRS + system and analyzed with ImageLab software (Bio-Rad, Hercules, CA, USA). All experiments were performed at least twice. DNA levels were quantified using SYBR Gold fluorescence. The total DNA amount was determined from the free DNA band at 0 nM SP1. The binding fraction was calculated using the following formula:


\begin{eqnarray*}
&& {\mathrm{Binding\ fraction\ of\ }}DN{{A}_{bound}}\ \left( {\mathrm{\% }} \right) =\nonumber\\ && \left[ {1 - \left( {\frac{{DN{{A}_{free}}}}{{DN{{A}_{total}}}}} \right)} \right] \times 100
\end{eqnarray*}

Fractions of bound DNA as a function of SP1 concentration from two separate experiments were fitted to the Hill equation using nonlinear regression in the GraphPad Prism software. The apparent *K*_D_ of SP1 for the DNA or RNA oligos was calculated as follows:


\begin{eqnarray*}
{\mathrm{Binding\ fraction\ of\ }}DN{{A}_{bound}} = \ \frac{{{{B}_{max}}\ \times \ {{X}^h}}}{{{{K}_D}^h + \ {{X}^h}}}
\end{eqnarray*}

where B_max_ is the maximum binding fraction of DNA_bound_, X is the protein concentration, and *h* is Hill's coefficient.

### Cell culture and luciferase reporter assay

The genomic region of the putative human Bcl3 promoter (chr19:45 251 800–45 252 003; 204 bp) was synthesized and cloned into the pGL4.11 luciferase reporter plasmid using NheI and HindIII restriction sites at the 5′ and 3′ ends, respectively. To determine the effect of G4 formation on promoter activity, mutant Bcl3 promoters listed in Table S1 were generated from the pGL4.11-WT plasmid via site-directed mutagenesis. For the luciferase assay, MDA-MB-231 cells were seeded in 24-well plates 1 day prior to transfection. On the day of transfection, 900 ng of the recombinant pGL4.11 plasmid and 100 ng of pRL-TK (internal control) were transiently transfected using TurboFect (Thermo Fisher Scientific, Waltham, MA, USA). For co-transfection experiments with pN3-SP1, which encodes the full-length human SP1 (UniProt ID: P08047), 450 ng of recombinant pGL4.11 plasmid and 50 ng of pRL-TK were co-transfected with 500 ng of either pN3 or pN3-SP1 plasmid. One day after transfection, a luciferase assay was performed using the Dual-Luciferase® Reporter Assay System (Promega, Madison, WI, USA). To assess the effect of G4-binding ligands TMPyP4 and PDS on promoter activity, the compounds were added to the culture medium at final concentrations of 10 or 20 μM. To evaluate the effect of RNA G4 on promoter activity, 0–500 ng of an RNA oligo derived from the Bcl3 transcript (chr19:45 252 319–45 252 336; 18 nt) was co-transfected with 450 ng of recombinant pGL4.11 and 50 ng of pRL-TK plasmid. All luciferase assays were performed in triplicate, starting from independent cell cultures.

### Confocal microscopic analysis of *in vitro* and *in vivo* SP1 condensation

For imaging SP1 condensation *in vitro*, full-length SP1 was labeled with Cy3-Maleimide (Click Chemistry Tools, Scottsdale, AZ, USA). SP1-Cy3 (final concentration: 455 nM) was incubated alone or with 45.5, 91, 227.5, or 455 nM dsBcl3-G4 in PS buffer (20 mM Tris-HCl, 100 mM KCl, 5 mM MgCl_2_, and 10% PEG8000). For co-localization analysis, dsBcl3-G4 labeled with Cy5 at the 5′ end of the positive strand was incubated with SP1-Cy3 at varying SP1-Cy3:dsBcl3-G4-Cy5 ratios (1:1 to 1:0.1), with SP1-Cy3 fixed at 455 nM. To examine the effect of RNA G4 on SP1 condensation, an RNA oligo derived from the *Bcl3* transcript (chr19:45 252 319–45 252 336; 18 nt) was annealed as described above and incubated with SP1 and dsBcl3-G4 at various DNA:RNA ratios in PS buffer. After 1 h of incubation, 4 μL of each sample was loaded onto a glass slide, covered with a coverslip, and imaged using a Zeiss LSM710 confocal microscope (Carl Zeiss, Oberkochen, Germany). To quantify droplet formation, 10 randomly selected fields were imaged per sample using identical exposure and ISO settings. Fluorescence intensities from TIFF images were analyzed using ImageJ software. The intensity threshold was set to 50, which corresponded to the near-maximal intensity observed in SP1-only samples, with other settings kept at default. Images with a mean fluorescence intensity exceeding 0.3 were considered indicative of condensation. The total droplet area was measured using ImageJ’s particle analysis tool. All experiments were performed at least twice. To visualize droplet fusion, samples were loaded into a custom chamber made by placing a coverslip over two parallel strips of double-sided tape on a glass slide and imaged as described above. For *in vivo* SP1 condensate imaging, an SP1-mCherry expression plasmid was constructed by cloning the full-length human SP1 sequence into the SOX2-T2A-mCherry plasmid (#127 538; Addgene, Watertown, MA, USA) using XbaI and KpnI restriction sites. MDA-MB-231 cells were seeded in 13 mm confocal dishes one day before transfection. On the day of transfection, 1 μg of SP1-mCherry plasmid was transiently transfected using TurboFect (Thermo Fisher Scientific, Waltham, MA, USA). Two days post-transfection, live-cell imaging was performed using a Zeiss LSM710 confocal microscope. For droplet quantification, five randomly selected fields containing approximately 10 cells each were imaged per sample under the same exposure time and ISO settings. TIFF images were analyzed using ImageJ to measure total droplet area, size, and count via the particle analysis tool.

### Fluorescence recovery after photo-bleaching (FRAP) assay

For the FRAP assay of SP1-Cy3 condensates *in vitro*, samples containing SP1 condensates with dsBcl3-G4 were prepared as described above and placed into a homemade chamber, where a coverslip was mounted on two parallel strips of double-sided tape on a glass slide. FRAP assays of SP1-Cy3 or SP1-mCherry condensates in cells were performed using a Zeiss LSM710 confocal microscope (Carl Zeiss, Oberkochen, Germany). A region of interest (ROI) was drawn within the condensates, a reference region was selected in a separate condensate within the nucleus, and a background region was defined in a non-condensate nuclear area. The ROI was photobleached after 50 iterations at 100% laser power. Images were acquired for 2 min at 1-s intervals. Following background subtraction and correction using intensity values from the background and reference regions, the ROI intensity was normalized to the pre-bleaching intensity. FRAP data from 15 droplets were fitted to a double-exponential function using GraphPad Prism software v8.3.0 (GraphPad Software, Boston, MA, USA).

### Immunofluorescence in cells

For immunofluorescence of endogenous SP1, Nucleolin (NCL), and RNA Polymerase II (RNA Pol II) in cells, MDA-MB-231 cells were subcultured in 13 mm confocal dishes 1 day prior to fixation. On the day of antibody treatment, cells were fixed in 4% formaldehyde in PBS for 10 min, followed by quenching with 0.1 M glycine in PBS for 10 min. Cells were then permeabilized with 0.1% Triton X-100 for 10 min and blocked with 1% BSA in PBS for 30 min. After extensive washing, Alexa Fluor 647-labeled SP1 antibody (#ab310048, Abcam, Cambridge, UK), Alexa Fluor 647-labeled NCL antibody (#sc-17826, Santa Cruz Biotechnology, Dallas, TX, USA), or Alexa Fluor 488-labeled RNA Pol II antibody (#ab309614, Abcam) was applied overnight at 4 °C at a 1:200 dilution To visualize G4 structures in cells, anti-DNA G4 antibody (BG4, Cat# MABE917, Merck, Rahway, NJ, USA) and FITC-labeled anti-FLAG antibody (Cat# A01632, GenScript, Piscataway, NJ, USA) were applied overnight at 4 °C at a 1:1000 dilution. To assess the effects of G4 ligands and RNA G4, cells were either treated with 10 or 20 μM of ligands or transfected with RNA oligos (20–100 nM), respectively, during culture. For negative controls, GFP with an N-terminal ER signal sequence was overexpressed. As a control for RNA G4 specificity, a non-G4 RNA was used in which all consecutive guanine nucleotides were removed. To quantify droplet formation, at least 10 randomly selected fields were imaged from each sample using identical laser power. The total droplet area, average size, and fluorescence intensity were measured using the particle analysis tool in ImageJ software. The intensity threshold was set to 55, corresponding to the average intensity of non-focal regions. Particle size was defined as 0.1– infinite, and circularity was set between 0.3 and 1.0.

### Site-directed mutagenesis for construction of SP1 deletion mutants

Domain deletion mutants of human SP1 were generated by site-directed mutagenesis using pN3-SP1 or SP1-mCherry plasmids as templates. The deleted regions in SP1 for each mutant are as follows: *Δ*IDR (1–93 amino acids, aa), *Δ*S/T1 (109–141 aa), *Δ*TAD A (146–251 aa), *Δ*S/T2 (329–395 aa), *Δ*TAD B (395–495 aa), *Δ*TAD C (496–610 aa), *Δ*DBD (626–708 aa), and *Δ*TAD D (708–785 aa). The primers used for site-directed mutagenesis PCR are listed in Supplementary Table S1.

### Confocal analysis of PP7-coating protein (PCP)-PP7 system

NLS-tdPCP-CFP was constructed by substituting GFP with CFP using AgeI and NotI restriction enzymes (#183 934; Addgene). For the pGL4.11-*Bcl3* promoter–12 × PP7 reporter plasmids, the 12 × PP7 sequence was amplified from pDZ617 pKAN 12 × PP7 (#72 237; Addgene) by PCR and cloned into the pGL4.11-*Bcl3* promoter reporter plasmid using the NcoI restriction enzyme. All primers used in this study are summarized in Supplementary Table S1. For confocal imaging, MDA-MB-231 cells were seeded in 13 mm confocal dishes 1 day prior to transfection. On the day of transfection, 400 ng SP1-mCherry, 300 ng NLS-tdPCP-CFP, and 300 ng pGL4.11-*Bcl3* promoter–12 × PP7 plasmids (1 μg total DNA) were transiently transfected using TurboFect (Thermo Fisher Scientific, Waltham, MA, USA). After 48 h, live cells were imaged using a Zeiss LSM710 confocal microscope (Carl Zeiss, Oberkochen, Germany). To assess the effect of RNA transfection, the same DNA plasmid combination (400 ng SP1-mCherry, 300 ng NLS-tdPCP-CFP, and 300 ng pGL4.11-*Bcl3* promoter–12 × PP7) was transfected one day before RNA transfection. On the day of RNA transfection, the culture medium was replaced, and 100 nM (final concentration) of either G4 or non-G4 RNA was transfected using TurboFect. Images were analyzed using ImageJ software. To quantify droplet formation, at least 10 randomly selected regions from each sample were imaged under identical laser power settings. The total droplet area, average size, and fluorescence intensity were measured using ImageJ’s particle analysis tool. The intensity threshold was set to 50–55, corresponding to the average intensity of nonfocal regions. Particle size was defined as 0.1– infinite, and circularity was set to 0.3–1.0.

### RNA extraction, quantitative real-time PCR (qRT-PCR), chromatin immunoprecipitation followed by qPCR (ChIP-qPCR), and western blot

Total RNA was reverse-transcribed using the RNA-to-cDNA EcoDry Premix (Oligo dT) kit (Takara, Kyoto, Japan). Quantitative reverse transcription PCR (qRT-PCR) was performed using iTaq™ Universal SYBR® Green Supermix (Bio-Rad, Hercules, CA, USA). Relative RNA expression levels were calculated using the *ΔΔ*Ct method For ChIP-qPCR analysis following SP1 overexpression or knockdown, MDA-MB-231 cells were subcultured in 24-well plates. After 1 day of culture, cells were transfected with either pN3-SP1 or siRNA targeting SP1 (Cat# AM16708, Thermo Fisher Scientific) using TurboFect. Following an additional day of culture, cells were fixed in 1% formaldehyde, quenched with glycine buffer, scraped into PBS containing 0.5 mM PMSF, and pelleted by centrifugation. Cells were lysed and homogenized, and nuclei were digested. Chromatin was sheared using an enzymatic cocktail (Active Motif, Cat# 53 014, Carlsbad, CA, USA). A portion of the sheared chromatin was reserved as input control. The remaining chromatin was incubated with anti-SP1 antibody (Cat# 07–645, Merck) and protein G magnetic beads for 16 h at 4°C. Beads were washed and incubated in elution and reverse cross-linking buffers at 95°C for 15 min. Proteinase K was added, and samples were incubated at 37°C for 1 h. The resulting solution was used directly for qPCR, performed with iTaq Universal SYBR Green Supermix (Bio-Rad). For ChIP-qPCR with the BG4 antibody (Cat# MABE917, Merck), MCF-7 and MDA-MB-231 cells were subcultured in 24-well plates. After 2 days of culture, cells were fixed with 1% formaldehyde, quenched with glycine buffer, and processed as above to prepare sheared chromatin. For ChIP-qPCR involving G4 ligands, MDA-MB-231 cells were subcultured in 24-well plates. After 1 day, 10 or 20 μM TMPyP4 or PDS was added to the culture medium, and cells were cultured for an additional day. Sheared chromatin was prepared as described above. All primers used for PCR are listed in Supplementary Table S1. For immunoblotting analysis of SP1 and BCL3 protein levels following siRNA-mediated knockdown, cells were cultured and treated with siRNAs as described above. Cell lysates were prepared by incubating with RIPA buffer (Merck, Rahway, NJ, USA) supplemented with protease inhibitor cocktail for 5 min at room temperature. Lysates were clarified by centrifugation, and equal amounts of total protein were resolved on a 12% SDS-PAGE gel. Proteins were transferred to PVDF membranes, which were blocked with 5% skim milk for 1 h at room temperature. Membranes were incubated with primary antibodies at 4°C overnight, followed by incubation with HRP-conjugated secondary antibodies for 1 h at room temperature. HRP signals were developed using Pierce™ ECL Western Blotting Substrate (Thermo Fisher Scientific), and images were acquired and analyzed using a ChemiDoc XRS + system (Bio-Rad).

### Transcript-mediated SP1 condensation inhibition and *in vitro* transcription

To investigate transcript-mediated inhibition of SP1 condensation, we constructed pGL4.11-*Bcl3* Promoter–*Bcl3* gene reporter plasmids containing the G4-forming region from the *Bcl3* gene (chr19:45 252 219–45 252 419; 201 bp), with either the WT or G6/8A promoter variant. For the *in vitro* transcription assay, 100 ng of reporter plasmid was incubated with 5 units of HeLa nuclear extract (Promega, Madison, WI, USA) in the presence or absence of SP1 at 30°C for 1 h. The resulting transcripts were analyzed by urea-PAGE. To evaluate the effects on SP1 condensation in live cells, MDA-MB-231 cells were seeded in 13 mm confocal dishes one day prior to transfection. On the day of transfection, a total of 1 μg plasmid DNA—comprising 400 ng SP1-mCherry, 300 ng NLS-tdPCP-CFP, and 300 ng pGL4.11-*Bcl3* Promoter–*Bcl3* gene reporter plasmid—was transiently transfected using TurboFect (Thermo Fisher Scientific, Waltham, MA, USA). After 48 h of incubation, live-cell imaging was performed using a Zeiss LSM710 confocal microscope (Carl Zeiss, Oberkochen, Germany). The CFP droplet area was quantified as described above.

## Results

### Identification of *Bcl3* promoter as a model of the SP1-enriched promoters containing G4 structure by applying the bioinformatics analyses

**Figure 1.**
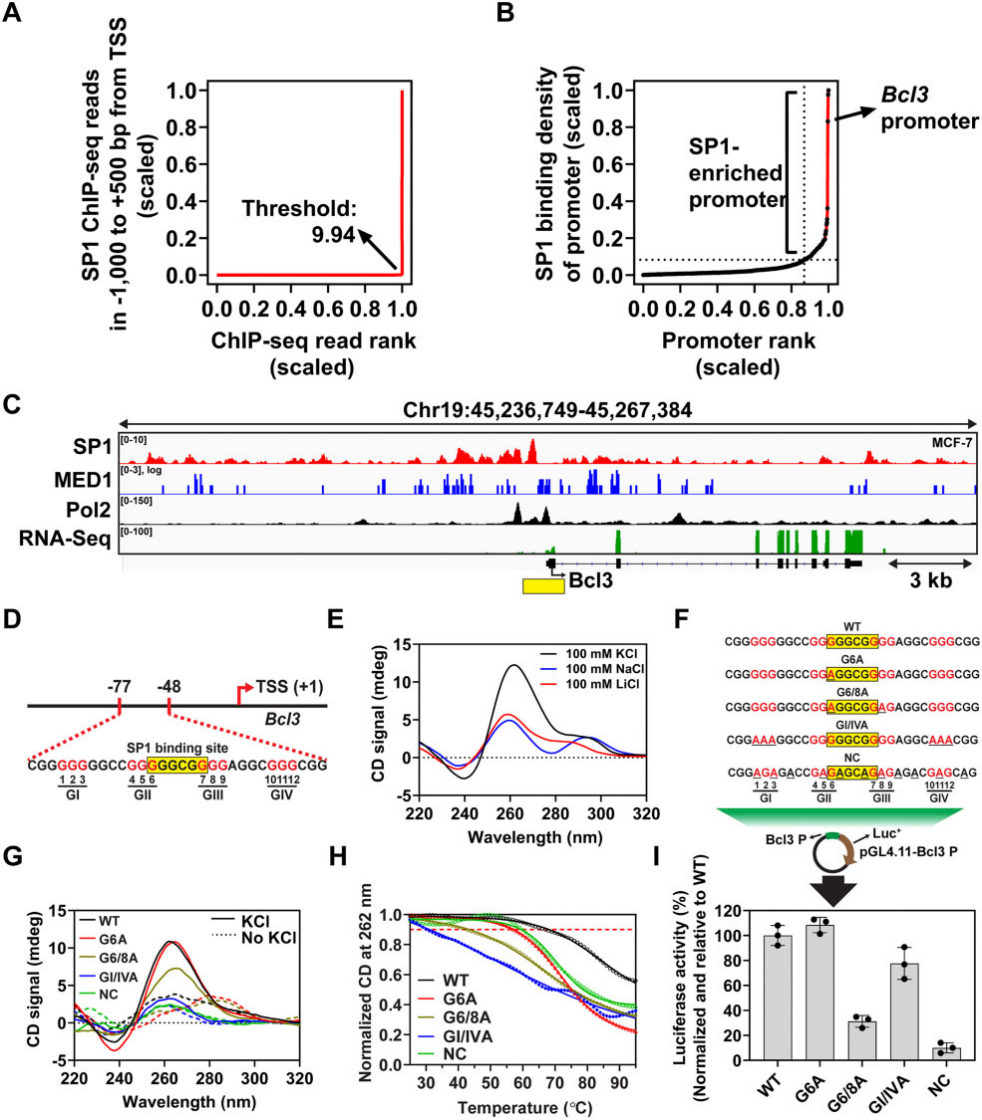
The Bcl3 promoter is SP1-enriched promoter and contains G-quadruplex (G4)-forming sequence. (**A**) A plot showing the distribution of SP1 ChIP followed by sequencing (ChIP-seq) reads (raw counts) across 46 447 gene promoters, ranked in ascending order. Both axes were scaled from 0 to 1. A geometric threshold value of 9.94 was determined, corresponding to a slope of 1.0 for the tangent line in the scaled plot. SP1 binding density for each promoter was defined as the proportion of SP1 ChIP-seq reads above this threshold relative to the total number of such reads. (**B**) A ranked plot of SP1 binding density for 545 promoters. Both axes were scaled from 0 to 1. Promoters lying above the line with a slope of 1 (tangent to the plot) were designated as SP1-enriched. (**C**) Genome browser (IGV) tracks showing ChIP-seq profiles of SP1, MED1, and RNA Polymerase II, as well as RNA-seq data around the *Bcl3* locus in MCF-7 cells. The promoter region spanning from −1000 to + 500 bp relative to the TSS is marked with a yellow bar. (**D**) Schematic representation of the G4-forming region within the Bcl3 promoter and its nucleotide sequence. (**E**) CD spectra of oligonucleotides from the *Bcl3* promoter annealed in the presence of 100 mM KCl, NaCl, or LiCl. (**F-H**) CD analysis of WT and mutant (MT) oligonucleotides, including G6A, G6/8A, GI/IVA, and a negative control (NC). (**F**) Sequences of WT and MT oligos. The putative SP1 binding site and guanines involved in G4 formation are highlighted. (**G**) CD spectra of WT and MT oligos annealed in the presence (solid lines) or absence (dashed lines) of KCl. (**H**) Thermal melting analysis of WT and MT oligos annealed in 100 mM KCl, recorded from 25°C to 95°C. (**I**) Luciferase reporter assay using pGL4.11 plasmids containing WT or MT *Bcl3* promoter sequences transfected into MDA-MB-231 cells. Luciferase activity was normalized to co-transfected Renilla luciferase from pRL-TK and presented relative to the WT promoter. Data represent the mean ± SD of at least three independent experiments. .

We hypothesized that promoters with higher SP1 binding capacity may facilitate SP1 condensation. To explore this, we identified SP1-enriched promoters in the MCF-7 human breast cancer cell line, in which SP1 plays a pivotal role in cancer-related processes [40]. Using publicly available SP1 ChIP-seq data from MCF-7 cells (GSM2423902), we calculated SP1 binding density across 46 447 gene promoters, defined as the region spanning –1000 bp to +500 bp from the transcription start site (TSS). SP1 binding density was determined by dividing the total number of SP1 ChIP-seq reads exceeding a defined threshold within each promoter by the total number of such reads across all promoters. The read threshold was determined geometrically as the point where a tangent line with a slope of 1 intersected the scaled plot of ChIP-seq read rank versus ChIP-seq read count (Fig. 1A). This threshold corresponded to a value of 9.94 on the scaled plot. Among all promoters analyzed, 545 contained at least one SP1 ChIP-seq read exceeding this threshold. These 545 promoters were ranked by SP1 binding density (Fig. 1B). Of these, 72 promoters positioned above the tangent line with a slope of 1 in the scaled plot were categorized as SP1-enriched, exhibiting an average SP1 binding density of 38.3. The remaining 473 promoters had an average SP1 binding density of 12.9. Notably, SP1-enriched promoters were predominantly associated with known oncogenes, including *Ahcyl2* [41], *Bcl3* [36, 42], and *Sp1* itself (Supplementary Fig. S1A) [26–28], suggesting that SP1’s oncogenic potential may arise from its role in regulating transcription of cancer-associated genes [43].

To investigate the impact of G4s on SP1 condensation and promoter activity, we further analyzed the 72 SP1-enriched promoters. G4-forming potential was assessed using QGRS Mapper, which identifies putative G4-forming sequences within DNA regions [44]. Among the 72 SP1-enriched promoters, 14 contained putative G4-forming sequences with G-scores exceeding 35—a threshold indicative of stable G4 formation [45] (Supplementary Fig. S1A). G4 formation in these promoters was validated by ChIP-seq analysis using the anti-G4 antibody BG4 in MCF-7 cells (GSM5501242) [46]. These 14 promoters are associated with genes involved in various biological processes, including cell signaling (*Ptpn12*, *Map2k1*, *Sp1*) [47–49]; cytoskeleton organization and cell motility (*Iqgap1*, *Cd2ap*, *Ypel2*) [50–52]; regulation of gene expression (*Bcl3*, *Gfi1b*, *Srrt*, *Sp1*) [53–56]; and cell growth and survival (*Ahcyl2*, *Akap1*, *Evi5l*, *Casc4*) [57–60]. Although the *Ahcyl2* and *Ptpn12* promoters rank higher than *Bcl3* in G4-forming potential, both contain multiple SP1-binding sites embedded within distinct G4-forming sequences, complicating the functional dissection of individual binding events. In contrast, the *Bcl3* promoter exhibits a simpler architecture, consisting of a single G4-forming sequence and a single SP1-binding site, allowing for more straightforward functional analysis. Accordingly, we selected the *Bcl3* promoter as our experimental model among the 14 SP1-enriched, G4-containing candidates, based on its high G4-forming potential (G-score = 42; ranked 3rd) and its established relevance to breast cancer [36, 42].

Genomic analysis using publicly available ChIP-seq data for SP1, MED1, and RNA polymerase II (RNA Pol II) in MCF-7 cells revealed that MED1 and RNA Pol II co-occupy the *Bcl3* promoter along with SP1, indicating that the *Bcl3* promoter is transcriptionally active in MCF-7 cells (Fig. 1C). Consistently, RNA-seq data confirmed active transcription of the *Bcl3* gene in MCF-7 cells (Fig. 1C). Similar genomic landscapes and active *Bcl3* transcription were also observed in other cancer cell lines, including lung adenocarcinoma (A549), lymphoblastoid (GM12878), and colorectal carcinoma (HCT-116), suggesting a shared regulatory mechanism governing *Bcl3* expression across diverse cancer types (Supplementary Fig. S1B). High occupancy of SP1, MED1, and RNA Pol II, along with active transcription, was also observed at other SP1-enriched promoters, such as those of the *Ahcyl2* and *Ptpn12* genes (Supplementary Fig. S1C). In contrast, promoters lacking SP1 enrichment, such as those of *Ddx4* and *Zap70*, showed minimal SP1 ChIP-seq signal and were devoid of these transcriptional and coactivator features, suggesting that SP1 specifically regulates a distinct subset of promoters (Supplementary Fig. S1D). To further investigate whether additional G4-binding TFs are involved at the *Bcl3* promoter, we analyzed publicly available ChIP-seq datasets for MAZ, NRF1, and E2F4 in MCF-7 cells. Among these, MAZ—a zinc-finger TF—displayed the strongest enrichment at the *Bcl3* promoter, with a binding pattern that closely overlapped with that of SP1 (Supplementary Fig. S2A). Notably, MAZ has been shown to form condensates at the *CCND1* promoter, suggesting that MAZ and SP1 may similarly co-condense at the *Bcl3* locus. This potential co-condensation could be facilitated by the similarity in their consensus binding motifs (SP1: 5′-GGGCGG-3′; MAZ: 5′-GGGTGGG-3′) [61].

To confirm the relationship between SP1 and the *Bcl3* gene, we assessed the effect of SP1 on *Bcl3* expression using RNA interference (RNAi). Under our experimental conditions, SP1 expression was reduced by approximately 50% at both the mRNA and protein levels following siRNA-mediated knockdown (Supplementary Fig. S2B and C). Following SP1 knockdown, *Bcl3* mRNA levels decreased to 55% of control levels, and *Bcl3* protein levels were reduced by approximately 40% compared to cells treated with non-targeting siRNA (Supplementary Fig. S2D and E). Although SP1 knockdown was partial, these results support a functional role for SP1 in the positive regulation of *Bcl3* transcription.

### Formation of G-quadruplex (G4) structure in the *Bcl3* promoter

Next, we validated the formation of G4 structures within the *Bcl3* promoter both *in vivo* and *in vitro*. To this end, we performed ChIP followed by quantitative PCR (ChIP-qPCR) using the BG4 antibody in two breast cancer cell lines, MCF-7 and MDA-MB-231. The *c-Myc* and *Esr1* promoters served as positive and negative controls, respectively, based on publicly available BG4 ChIP-seq data from MCF-7 cells [46]. Consistent with previous ChIP-seq results, we observed strong BG4 enrichment at the *Bcl3* promoter in both cell lines (Supplementary Fig. S3A and B). Under the same conditions, the BG4 antibody also bound to the *c-Myc* promoter, whereas binding at the *Esr1* promoter was markedly reduced. For *in vitro* G4 formation analysis, CD spectroscopy, ThT fluorescence assay, and DMS footprinting were performed. A 30-nt oligonucleotide derived from the *Bcl3* promoter (chr19:45 251 889–45 251 918) was denatured at 95°C and gradually cooled to 25°C to facilitate G4 structure formation (Fig. 1D). CD analysis of the oligo annealed with 100 mM KCl revealed characteristic negative and positive peaks at 240 and 260 nm, respectively, along with a subtle shoulder near 290 nm (Fig. 1E). This finding suggests that the oligo predominantly adopts a parallel G4 structure, with a minor contribution from a hybrid G4 structure as well [62]. Notably, neither LiCl nor NaCl, nor the absence of KCl, induced a parallel G4 structure (Fig. 1E and Supplementary Fig. S3C). ThT, a fluorescent sensor for G4 structures [63], exhibited a strong emission peak at 490 nm with the G-rich positive strand of the 30-nt oligonucleotide, compared to the C-rich negative strand, confirming G4 formation in the G-rich sequence (Supplementary Fig. S3D). DMS footprinting revealed low-efficiency cleavage of guanine residues by piperidine in the presence of KCl, indicating protection due to G4 structure formation within the *Bcl3* promoter (Supplementary Fig. S3E). Collectively, these results confirm G4 structure formation within the *Bcl3* promoter both *in vitro* and *in vivo*.

### Effect of G4 formation in the *Bcl3* promoter on transcriptional regulation

To investigate the role of G4 formation in the *Bcl3* promoter in transcriptional regulation, we conducted luciferase reporter assays using pGL4.11 reporter plasmids containing the *Bcl3* promoter region (chr19:45 251 800–45 252 003; 204 bp) (Fig. 1F). Mutant sequences (MTs) were designed as controls, incorporating G-to-A substitutions either within the G-tracts or the SP1-binding site (Fig. 1F). CD analysis of WT and MT oligonucleotides revealed that the G6A mutant (with the 6th guanine substituted with adenine) retained a parallel G4 structure similar to the WT, whereas the G6/8A mutant (with two G-to-A substitutions) disrupted G4 formation (Fig. 1G). Substituting all guanines in the GI and GIV tracts with adenines (GI/IVA) significantly inhibited G4 formation (Fig. 1G). The negative control (NC) oligo, which lacked both the G-tract and SP1-binding site, exhibited no detectable secondary structure formation (Fig. 1G). All oligos showed poor G4 formation in the absence of KCl (Fig. 1G, dashed lines). The thermal stability of the G4 structures was further assessed by monitoring CD signal changes over increasing temperatures. Since the CD melting curves did not yield reliable fits for calculating *T*_m_ values, we instead compared the temperatures at which the CD signals decreased to 90% of their initial maximum values. Based on this analysis, the CD signal of the WT G4 dropped to 90% at 68°C, while the G6A mutant dropped at 58°C. In contrast, the G6/8A and GI/IVA mutants showed significantly lower thermal stability, with 10% signal loss occurring at 43°C and 31°C, respectively (Fig. 1H). The formation of G4 structures in both WT and MT oligos was further validated through UV melting analysis at 295 nm. The results showed sigmoidal decreases in UV absorbance for both WT and G6A oligos as the temperature increased (Supplementary Fig. S3F). This sigmoidal behavior indicates cooperativity in the melting process, a characteristic feature of G4 structures [64]. Overall, the results from both CD and UV melting analyses demonstrate the impact of each mutation on the formation and stability of G4 structures in the *Bcl3* promoter *in vitro*.

For luciferase reporter assays, MDA-MB-231 cells were transfected with pGL4.11 reporter plasmids containing the *Bcl3* promoter with either WT or mutant (MT) sequences. The plasmid containing the G6A mutation exhibited luciferase activity comparable to that of the WT (Fig. 1I). In contrast, the G6/8A-containing plasmid exhibited only 31% of the luciferase activity observed with the WT construct. Notably, the GI/IVA-containing plasmid retained 77% of WT activity, suggesting that the GI/IVA mutant may still adopt a G4 structure under double-stranded DNA conditions. The NC reporter displayed the lowest luciferase activity among all constructs (Fig. 1I). Collectively, these results reveal a strong correlation between promoter activity and G4 structure formation or stability within the *Bcl3* promoter, underscoring the critical role of G4 elements in transcriptional regulation.

To assess the impact of G4 structures on *Bcl3* transcriptional regulation, we used the G4-binding ligands TMPyP4 and PDS to modulate G4 formation within the *Bcl3* promoter. For CD analysis, oligonucleotides were annealed in the presence of 20 μM ligand. In WT G4, TMPyP4 slightly reduced the CD signal near 284 nm and narrowed the 264 nm peak, suggesting a more conformationally homogeneous structure (Supplementary Fig. S4A). In contrast, in MT G4s, TMPyP4 markedly decreased the CD signal near 264 nm and increased the signal near 284 nm, indicative of a structural transition toward basket-type G4 conformations (Supplementary Fig. S4A) [62]. Conversely, PDS maintained or increased the signals at 264 nm in both WT and MT G4s, suggesting stabilization of parallel G4 formation (Supplementary Fig. S4A). We then compared the thermal stability of WT and MT G4s in the presence or absence of G4-binding ligands. For MT G4s treated with TMPyP4, the CD spectral maxima were observed at 284 nm (Supplementary Fig. S4A); therefore, CD signals were normalized to the ellipticity at 284 nm. In contrast, for samples treated with PDS, the spectral maxima remained at 264 nm, and normalization was performed accordingly at this wavelength. Thermal stability analysis revealed a decrease in CD signal at approximately 55°C for the WT control, 45°C for TMPyP4-treated WT, and 75°C for PDS-treated WT (Supplementary Fig. S4B). Both TMPyP4 and PDS increased G4 stability in all MT G4s compared to their respective controls, with PDS exhibiting a greater stabilizing effect (Supplementary Fig. S4B). We further assessed the effects of G4-binding ligands on *Bcl3* promoter activity using luciferase reporter assays with plasmids containing either WT or MT *Bcl3* promoter sequences. Treatment with 10 μM TMPyP4 led to a 63% reduction in luciferase activity driven by the WT promoter (Supplementary Fig. S4C). In contrast, TMPyP4 significantly increased luciferase activity from the mutant promoters: G6A (164%), G6/8A (253%), and GI/IVA (221%) relative to their respective untreated controls (Supplementary Fig. S4D). Similarly, treatment with PDS enhanced luciferase activity across all constructs, with increases of 131% (WT), 215% (G6A), 343% (G6/8A), and 177% (GI/IVA) compared to untreated controls. These results indicate that G4 formation within the *Bcl3* promoter promotes transcription and that this activity can be differentially modulated by G4-binding ligands in both positive and negative directions depending on the sequence context and the ligand used.

### G4 regulates the binding of SP1 to the *Bcl3* promoter

We then investigated SP1 binding to the *Bcl3* promoter. Using publicly available SP1 ChIP-seq data from MCF-7 cells (GEO: GSM2423902) [65], we selected the *Sp1* and *Rinl* promoters as positive and negative controls, respectively, for our ChIP-qPCR analysis. Consistent with the reference dataset, we observed strong SP1 occupancy at both the *Bcl3* and *Sp1* promoters in MDA-MB-231 cells, whereas the *Rinl* promoter showed negligible SP1 binding, comparable to the IgG control (Supplementary Fig. S5A). To further investigate SP1 binding to the G-rich sequence within the *Bcl3* promoter, where G4 formation was previously demonstrated, we performed EMSA to assess SP1 binding affinities. For EMSA, we utilized a 24-nt oligo (chr19:45 251 892–45 251 915) that excludes the flanking CGG sequences present in the 30-nt oligo used for spectroscopic assays, in order to minimize potential contributions from these regions to G4 formation. Notably, the 24-nt oligos exhibited CD spectra nearly identical to those of the 30-nt versions (Supplementary Fig. S5B), indicating that the core G4 structure is preserved. To further test G4-dependence, we introduced the PCNG4 oligo, which lacks successive guanines except within the SP1-binding motif to prevent G4 formation (Supplementary Fig. S5C). On a 20% polyacrylamide gel, the WT oligonucleotide in the presence of KCl exhibited both higher- and lower-mobility bands compared to the WT in the absence of KCl, indicating the formation of inter- and intramolecular G-quadruplexes (G4^Inter^ and G4^Intra^), respectively. In contrast, the oligonucleotide predominantly existed as single-stranded DNA (ssDNA) in the absence of KCl (Supplementary Fig. S5D). However, neither the GI/IVA nor the NC oligonucleotides exhibited any mobility shift upon KCl treatment, suggesting that, unlike the WT, they do not undergo KCl-dependent conformational changes (Supplementary Fig. S5D). Since PCNG4 formed intermolecular and non-G4 species in the presence of KCl, we separated these by size-exclusion chromatography and analyzed their structures using CD spectroscopy. The higher (Inter 1) and intermediate (Inter 2) migration fractions exhibited CD signatures of parallel G4s, with a positive peak at 260 nm and a negative peak at 240 nm (Supplementary Fig. S5E). In contrast, the lowest molecular weight fraction—migrating similarly to ssDNA—lacked G4-specific CD signals, consistent with an unstructured single-stranded state. Accordingly, the purified PCNG4 Inter 1 fraction was used for the SP1 binding analysis. For the WT oligo, we proceeded without further purification, as we did not detect non-G4 species under the same conditions (Supplementary Fig. S5D).

**Figure 2.**
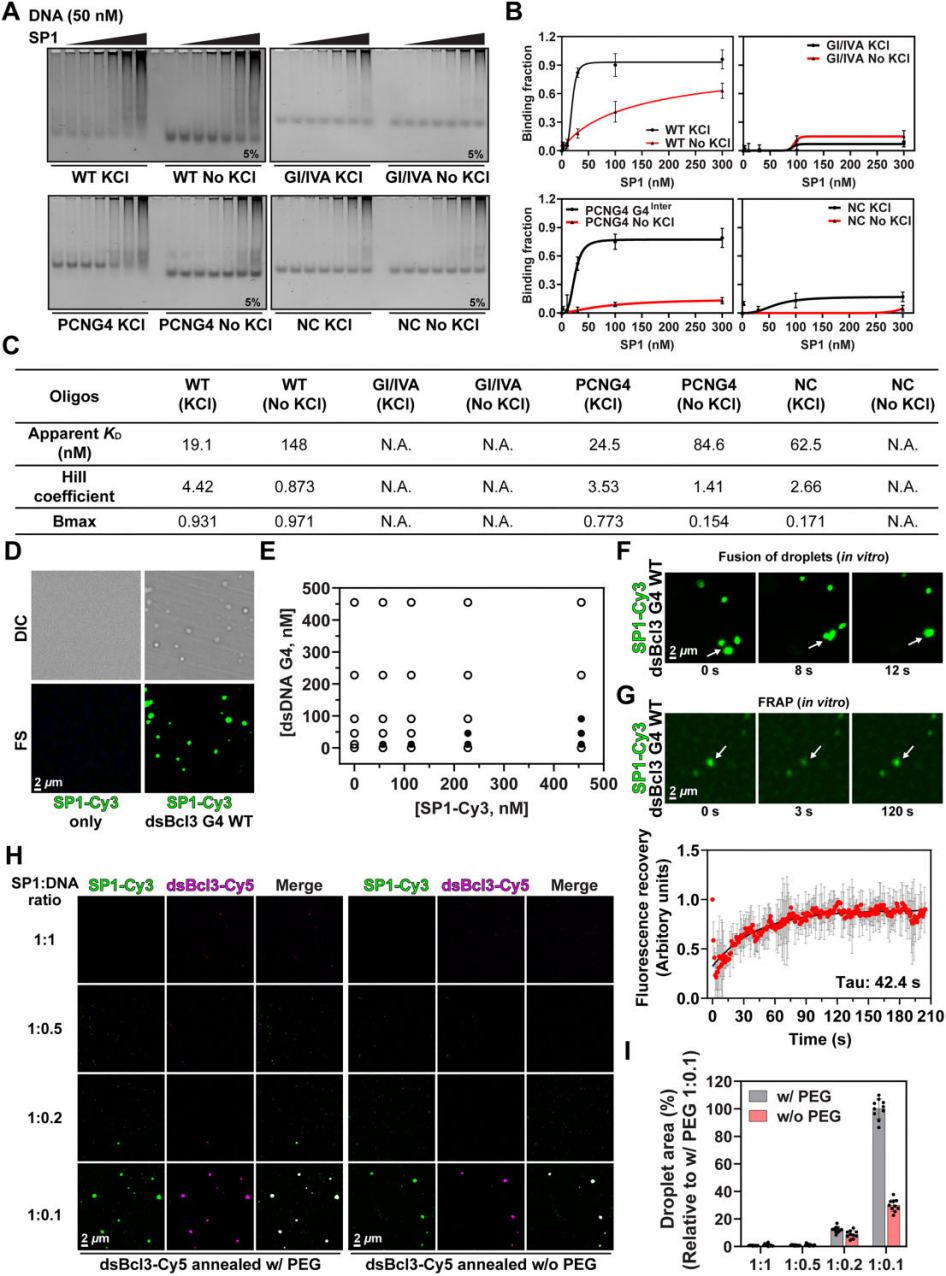
G4 facilitates the binding of SP1 to DNA and the condensation of SP1 with DNA. (**A**) EMSA of SP1 binding to WT, GI/IVA, PCNG4, and negative control (NC) oligonucleotides. Increasing concentrations of SP1 were incubated with each oligo and resolved on a 5% polyacrylamide gel. Bands migrating more slowly than free oligo were interpreted as SP1-bound DNA complexes. (**B**) Quantification of binding fractions for each oligo across varying SP1 concentrations, derived from the EMSA in (A). Binding fraction was calculated using the formula: 1 – (free DNA intensity / free DNA intensity at 0 nM SP1). Data represent mean ± standard deviation from three independent experiments. (**C**) Apparent dissociation constant (*K*_D_), Hill coefficient and Bmax values were obtained by fitting the binding curves in (B) to the Hill equation using nonlinear regression in GraphPad Prism. (**D**) Phase separation of SP1-Cy3 with or without double-stranded *Bcl3* G4 oligo (dsBcl3). Condensates were visualized by differential interference contrast (DIC) and fluorescence (FS) microscopy. (**E**) Phase diagram indicating condensate formation across varying concentrations of SP1 and dsBcl3. Filled and open circles represent conditions that did or did not produce condensates, respectively. Condensate formation was defined by a mean FS intensity > 0.3, as quantified using ImageJ. (**F**) Representative FS images of SP1-Cy3/dsBcl3 G4 droplets showing fusion events, indicating liquid-like behavior *in vitro*. (**G**) FRAP analysis of SP1-Cy3/dsBcl3 G4 condensates. Bleaching was initiated at 0 s. The recovery curve shows the mean and standard deviation of Cy3 intensity across 15 droplets. Data were fitted to a double exponential function. (**H**) FS images of SP1-Cy3 condensates formed at varying SP1:dsBcl3-Cy5 ratios. dsBcl3-Cy5 was pre-annealed in the presence or absence of KCl. (**I**) Quantification of total condensate area relative to that at an SP1:DNA ratio of 1:0.1 (with KCl). Data represent the mean ± standard deviation from ten randomly selected fields of view. The graphs show the mean and standard deviation of ten randomly chosen regions. Experiments were performed independently twice.

SP1 binding to WT (KCl) yielded an apparent dissociation constant (*K*_D_) of 19.1 nM (Fig. 2A–C), in line with prior reports (23–32 nM) [66, 67]. In contrast, binding to WT oligos annealed without KCl produced a *K*_D_ of 148 nM, confirming preferential SP1 interaction with G4-containing DNA. Similarly, SP1 binding to G4-deficient oligos (GI/IVA and NC) was substantially reduced. Notably, SP1 bound to PCNG4 Inter 1 with an apparent *K*_D_ of 24.5 nM, comparable to that of WT G4 (Fig. 2A–C), while it bound poorly to PCNG4 annealed without KCl, supporting the role of G4 structure in enhancing SP1 binding. Some non-specific binding of SP1 to NC oligos was also observed, indicating that SP1 engages both specific and non-specific interactions with DNA (Fig. 2A–C). Furthermore, EMSA revealed elevated Hill coefficients for SP1 binding to WT G4 (KCl, Hill coefficient = 4.42) and PCNG4 Inter 1 (Hill coefficient = 3.53), compared to lower values for non-G4 oligos (range: 0.873–2.66) (Fig. 2C). These results suggest that G4 structures promote cooperative SP1 binding, and the high Hill coefficients support the presence of multivalent interactions.

To complement the EMSA results, we performed biolayer interferometry (BLI) using biotinylated WT or mutant oligonucleotides immobilized on a streptavidin-coated sensor chip. The same oligos used in EMSA were employed to ensure consistency in binding comparisons. We calculated the kinetic parameters of SP1 binding to G4 and non-G4 oligonucleotides using 457 nM SP1 by fitting the binding curves to a local 1:1 binding model (Supplementary Fig. S6A). Under these conditions, SP1 exhibited a local *K*_D_ of 2–4 nM for oligos annealed in KCl (Supplementary Fig. S6A and B). In contrast, the local *K*_D_ was 6–8 nM for oligos annealed without KCl, indicating increased SP1 binding affinity upon G4 formation. The association rates (*k*_a_) of SP1 with oligos annealed in KCl (2.27–2.88 × 10^5^ M−^1^s−^1^) were comparable to those with oligos annealed without KCl (2.46–3.39 × 10^5^ M−^1^s−^1^), suggesting that the lower local *K*_D_ for the KCl-annealed oligos resulted from slower SP1 dissociation rates (*k*_d_) (Supplementary Fig. S6A and B). These BLI-derived *K*_D_ values are notably lower than those observed by EMSA (19.1 nM), likely due to BLI capturing both primary interactions between SP1 and immobilized DNA, and secondary interactions between DNA-bound SP1 and free SP1 in solution, as reported in the previous study [31]. Supporting this interpretation, SP1 binding increased linearly with concentration without reaching saturation, as shown in Supplementary Fig. S6C and D. Notably, a steeper slope was observed for WT (KCl) (0.22), compared to WT (no KCl) and GI/IVA (KCl) (both slope = 0.12), underscoring the role of G4 structures in facilitating high-affinity and multivalent SP1 binding. To further support the multivalent SP1 binding to DNA, we fitted the binding curves using a 2-step binding model [68]. This model yielded improved *R*^2^ values, particularly at higher SP1 concentrations, suggesting that SP1 interacts with DNA in a multivalent manner rather than through a simple 1:1 binding mode (Supplementary Fig. S6E and F).

### *Bcl3* promoter facilitates SP1 condensation in a G4-dependent manner

We next investigated SP1 condensation in the presence of DNA and assessed the impact of G4 structures on this process. To mimic *in vivo* conditions, we used a 90-bp double-stranded DNA fragment from the *Bcl3* promoter region, comprising a 30-bp G4-forming sequence flanked by 30-bp regions on both sides (dsBcl3; chr19:45 251 859–45 251 948). NMM, which preferentially binds to parallel and hybrid G4 structures [38], was used to confirm G4 formation in the *Bcl3* promoter. As molecular crowding induced by PEG stabilizes G4s in dsDNA [38, 69], G4 formation was tested using 40% PEG200 or 10% PEG8,000. NMM fluorescence near 610 nm was observed when WT dsBcl3 was annealed with 1 mM KCl in the presence of 10% PEG8,000, and with 100 mM KCl in the absence of PEG8,000 (Supplementary Fig. S7A, left). However, under the same conditions, the GI/IVA and G6/8A dsBcl3 mutants did not exhibit NMM fluorescence peaks, suggesting an absence of detectable G4 formation (Supplementary Fig. S7A, middle and right). We further evaluate G4 formation in WT and mutant dsBcl3 constructs, we performed CD spectroscopy and atomic force microscopy (AFM) analyses. In Tris-EDTA (TE) buffer containing 1 mM KCl (“No PEG + 1 mM KCl” condition), all three constructs—WT, GI/IVA, and G6/8A—exhibited CD spectra characteristic of canonical B-form dsDNA, with a positive peak at 280 nm and a negative peak at 240 nm (Supplementary Fig. S7B). Under molecular crowding conditions (10% PEG 8 000 with 1 mM KCl), WT dsBcl3 displayed a distinct CD signature with positive peaks at 265 and 290 nm and a negative peak at 240 nm, consistent with hybrid G4 formation. Interestingly, under 100 mM KCl with PEG, the WT construct exhibited only a single positive peak at 265 nm, suggesting a conformational shift toward alternative secondary structures, such as hairpins (Supplementary Fig. S7B, WT) [70]. The GI/IVA mutant exhibited a CD spectrum indicative of an antiparallel G4 structure—marked by a negative peak near 260 nm and a positive peak at 295 nm—under 100 mM KCl with PEG (Supplementary Fig. S7B, GI/IVA). In contrast, the G6/8A mutant showed no significant spectral changes under any salt or crowding condition, consistent with a lack of G4-forming potential (Supplementary Fig. S7B, G6/8A).

AFM analysis corroborated these observations. Under 10% PEG 8000 and low-salt conditions, WT dsBcl3 formed a prominent central G4 structure (Supplementary Fig. S7C, WT), whereas neither GI/IVA nor G6/8A formed discernible G4 structures under the same conditions (Supplementary Fig. S7C, GI/IVA and G6/8A). Under 100 mM KCl with PEG, WT dsBcl3 exhibited secondary structures at both ends of the molecule, with distinct heights differing from the central G4 structure seen under low-salt conditions (Supplementary Fig. S7D and E). Based on the NMM analysis and sequence context, these end-associated structures are likely non-G4 structure. Under identical conditions, GI/IVA dsBcl3 exhibited a central protrusion with a height similar to that of WT G4 structures, supporting G4 formation. This observation may explain the relatively high luciferase activity of the GI/IVA reporter in Fig. 1I (Supplementary Fig. S7B and D). In contrast, G6/8A dsBcl3 displayed no such protrusions, further confirming its lack of G4-forming capability. Together, these results demonstrate that WT dsBcl3 forms stable G4 structures under salt-deficient and molecular crowding conditions, resembling telomeric G4s [71].

We next examined the effect of the G4 structure in WT dsBcl3 on SP1 condensation by incubating WT dsBcl3 with Cy3-labeled SP1 (SP1-Cy3) in the presence of 10% PEG8,000. SP1-Cy3 droplets formed only when the WT dsBcl3 G4 structure was present (Fig. 2D), and their formation was influenced by the concentrations of SP1 and dsBcl3, as well as by the salt concentration (Fig. 2E and Supplementary Fig. S8A). In the case of SP1 condensation with WT dsBcl3 G4, we observed maximal condensation at 100 mM KCl (Supplementary Fig. S8A), even though nearly maximal G4 formation was achieved at 10 mM KCl concentration (Supplementary Fig. S3C). This indicates that while G4 structures can form efficiently at 10 mM KCl, the optimal biophysical environment for SP1 condensation may require higher salt concentrations. These findings are consistent with prior reports showing that phase separation of DNA-binding proteins is modulated by factors beyond G4 stability [17, 72, 73]. The droplets demonstrated fusion behavior (Fig. 2F). Furthermore, a fluorescence recovery after photobleaching (FRAP) assay was performed to assess the diffusion of unbleached molecules from the surrounding environment into the bleached region. The results showed that SP1 condensates exhibited fluorescence recovery with a Tau of 42.4 s after photobleaching (Fig. 2G). The observed fluorescence recovery in the FRAP assay reflects the dynamic exchange of SP1 molecules between the condensate and the surrounding environment, consistent with diffusion-driven recovery. This behavior is characteristic of liquid-like condensates and supports the interpretation that SP1 condensates are dynamic and reversible. To further assess the impact of G4 structures on SP1 condensation with DNA, we examined SP1 condensation using Cy5-labeled WT dsBcl3 in the presence or absence of 10% PEG8,000, across a range of SP1:DNA ratios. We found that SP1 condensation is highly influenced by G4 DNA concentration, with only 10% SP1 condensation observed at an SP1:G4 DNA ratio of 1:0.2, compared to 1:0.1 (Fig. 2H and I). Additionally, 1,6-Hexanediol (1,6-HD), a small molecule known to disrupt hydrophobic interactions in biomolecular condensates, significantly reduced SP1 condensation (Supplementary Fig. S8B, left). This observation underscores the importance of hydrophobic interactions in SP1 phase separation. Furthermore, 1,6-HD treatment also diminished the intense, immobile signals retained in the gel wells during EMSA (Supplementary Fig. S8B, right), supporting the interpretation that these signals represent SP1-G4 condensates that are disrupted by 1,6-HD. SP1 condensation with PEG-annealed dsBcl3 was significantly greater than that observed without PEG, indicating that SP1 condensation is G4-dependent (Fig. 2H and I). Consistent with this, similar results were obtained when comparing WT and mutant dsBcl3 (Supplementary Fig. S8C–F). Furthermore, SP1 also formed condensates with the dsDNA promoters of *Ahcyl2* (dsAHCYL2) and *Ptpn12* (dsPTPN12), which rank first and second among G4-harboring, SP1-enriched promoters (Supplementary Fig. S9A and B). These condensates formed when the promoters were annealed in the presence of PEG8,000, highlighting both the versatility of SP1 condensation across various G4-containing promoters and the critical role of G4 formation in facilitating SP1–DNA condensation.

**Figure 3.**
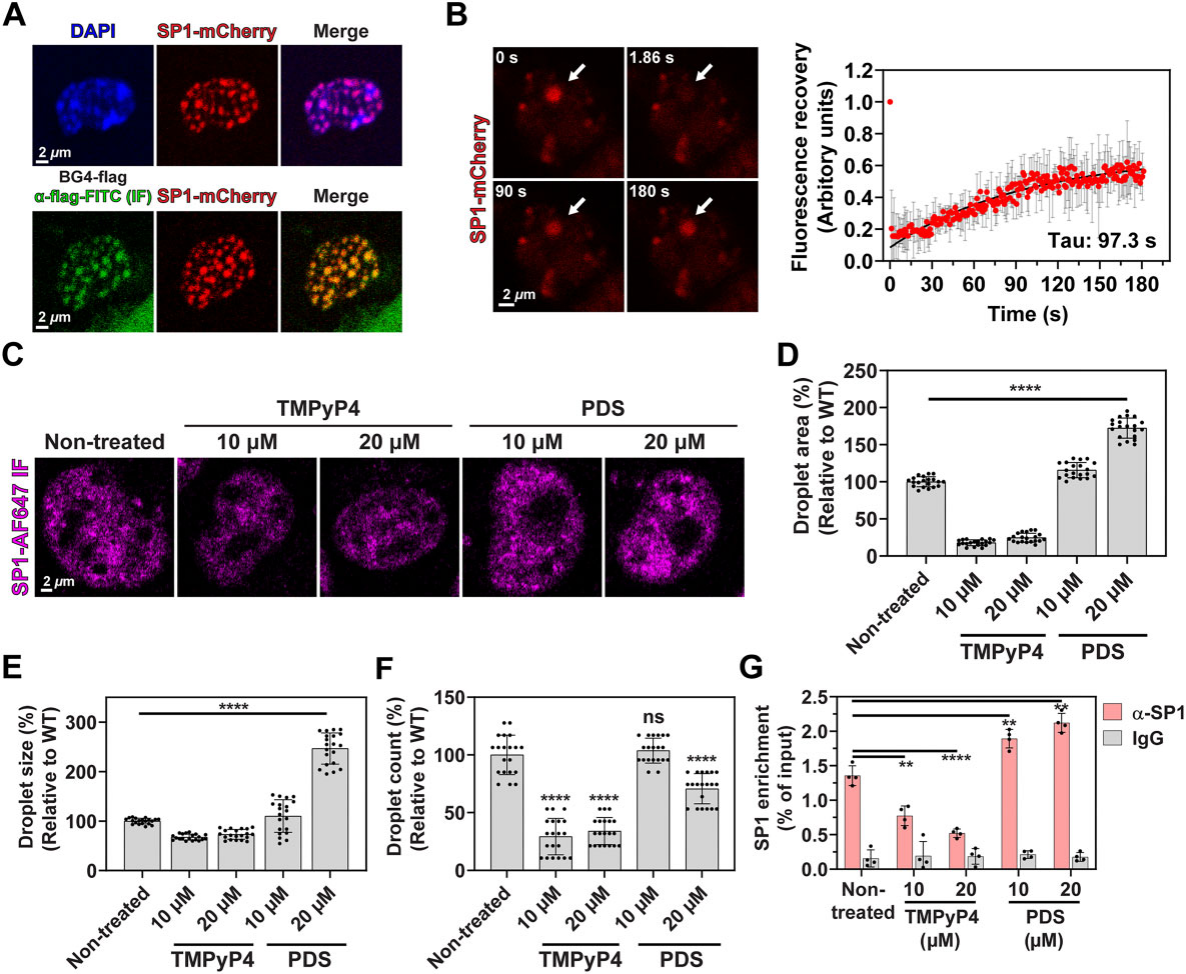
G4 ligands modulate SP1 condensation in cells. (**A**) Representative FS images of MDA-MB-231 cells expressing SP1-mCherry (top) and immunofluorescence (IF) images of cells stained with FITC-labeled BG4 antibody (bottom), which recognizes G4 structures. Images were captured at 40× magnification. Enlarged views of single cells are shown. (**B**) FRAP analysis of SP1-mCherry condensates. Photobleaching was initiated at 0 s. The recovery curve shows the mean and standard deviation of mCherry intensity across 15 condensates. The curve was fitted using a double exponential function. (**C-F**) IF analysis of endogenous SP1 using Alexa Fluor 647-conjugated anti-SP1 antibody in MDA-MB-231 cells treated with TMPyP4 or PDS at 10 or 20 μM. (**D**) Quantification of total condensate area, (**E**) average droplet size, and (**F**) droplet count, each shown relative to the untreated control. Data represent the mean ± standard deviation from ten randomly selected regions. Experiments were independently performed twice. (**G**) ChIP followed by quantitative PCR (ChIP-qPCR) analysis of SP1 binding at the *Bcl3* promoter in MDA-MB-231 cells treated with TMPyP4 or PDS. An anti-SP1 antibody was used for immunoprecipitation, and normal IgG served as a negative control. Values are normalized to 5% input. Data represent mean ± standard deviation from at least three independent experiments.

To investigate SP1 condensation *in vivo*, we overexpressed SP1-mCherry in MDA-MB-231 cells and observed the formation of nuclear mCherry foci (Fig. 3A, top). Previous studies have reported the presence of G4 structures within phase-separated droplets in cells [74]. To investigate whether SP1 co-condenses with G4 structures in cells, we performed immunofluorescence (IF) analysis using the BG4 antibody in combination with a FITC-labeled anti-Flag antibody. In cells treated with BG4 alone, distinct nuclear BG4 foci were observed, consistent with previous reports (Supplementary Fig. S10A, left) [74, 75]. When BG4 staining was performed in SP1-mCherry–expressing cells, we observed substantial co-localization of SP1 with BG4 foci, indicating co-condensation of SP1 with G4 structures (Fig. 3A, bottom). Similarly, BG4 foci showed strong co-localization with Alexa Fluor 647–labeled nucleolin (NCL), a known G4-binding protein (Supplementary Fig. S10A, middle) [76]. In contrast, no overlap was detected between BG4 foci and mCherry-labeled SOX2, a TF that binds AT-rich motifs (Supplementary Fig. S10A, right), supporting the specificity of BG4 signal for G4-associated proteins. FRAP analysis confirmed the liquid-like properties of SP1-mCherry foci, with a recovery half-time (Tau) of 97.3 s (Fig. 3B). To determine whether G4 structures influence SP1 condensation in cells, we treated MDA-MB-231 cells with TMPyP4 or PDS. SP1 condensates were visualized by immunofluorescence using an Alexa Fluor 647-conjugated antibody (anti-SP1-AF647) (Fig. 3C). Distinct AF647-labeled foci were detected, indicating the presence of endogenous SP1 within condensates (Fig. 3C, Non-treated). TMPyP4 treatment reduced the total area of SP1 condensates, with only minor effects on droplet size (Fig. 3C–F, TMPyP4), whereas PDS treatment significantly increased both the total area and size of the condensates (Fig. 3C–F, PDS). As a negative control, GFP with an N-terminal ER signal sequence exhibited no apparent change in the total area of GFP foci following treatment with either TMPyP4 or PDS, indicating that these compounds did not affect the behavior of the control protein (Supplementary Fig. S10B and C). Furthermore, qPCR analysis showed that SP1 occupancy at the *Bcl3* promoter decreased following TMPyP4 treatment and increased with PDS treatment (Fig. 3G). Given the effects of these G4 ligands on G4 structure in the *Bcl3* promoter, as demonstrated by CD analysis (Supplementary Fig. S4), these findings support a regulatory role for G4 structures in modulating SP1 condensation dynamics.

### G4-dependent SP1 condensation promotes transcription

**Figure 4.**
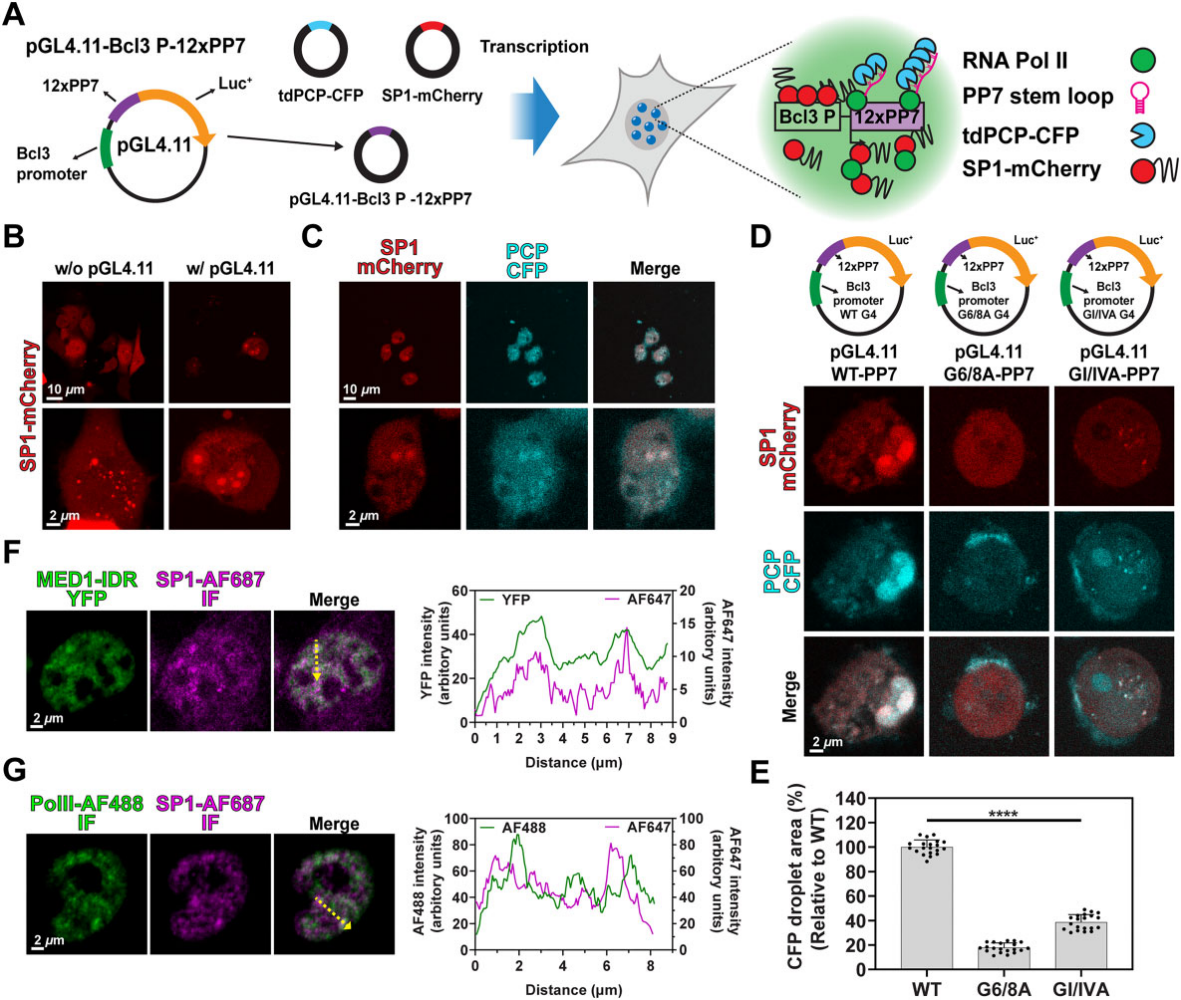
G4-dependent SP1 condensation enhances transcription. (**A**) Schematic of the pGL4.11-*Bcl3*-12 × PP7 reporter plasmid (left) and the PP7–PCP system used to visualize nascent transcripts in living cells (right). (**B**) FS images of MDA-MB-231 cells transfected with SP1-mCherry alone (without reporter plasmid; w/o pGL4.11) or co-transfected with the pGL4.11-*Bcl3*-12 × PP7 reporter plasmid (with reporter; w pGL4.11). Top panels show 40 × magnification images; bottom panels display close-up images of individual cells. (**C**) Representative FS images of MDA-MB-231 cells co-transfected with SP1-mCherry, PCP-CFP, and the reporter plasmid. Top: 40 × magnification images; bottom: enlarged views of single cells. (**D**) Schematic of reporter plasmid constructs containing WT, G6/8A, or GI/IVA mutant *Bcl3* promoter sequences (top). Representative FS images of MDA-MB-231 cells co-transfected with SP1-mCherry, PCP-CFP, and the respective reporter plasmid (bottom). Images were acquired at 40 × magnification, and single-cell close-ups are shown. (**E**) Quantification of total PCP-CFP condensate area, normalized to the signal from cells transfected with the WT reporter construct. Graphs represent the mean ± standard deviation from ten randomly selected fields. Experiments were independently repeated twice. (**F, G**) Colocalization of SP1 with transcriptional coactivators in MDA-MB-231 cells. (**F**) IF of SP1-AF647 in cells expressing MED1-IDR-YFP. Line profile analysis of fluorescence intensity along the indicated black dashed line is shown on the right. (**G**) Dual IF of SP1-AF647 (magenta) and RNA Polymerase II-AF488. Line profile along the yellow dashed line shows spatial overlap of fluorescence signals (right).

To further investigate the link between SP1 condensation and transcriptional regulation of the *Bcl3* promoter, we first assessed the effect of SP1 overexpression on *Bcl3* promoter activity using a luciferase reporter assay. The results demonstrated that SP1 overexpression enhanced the activity of the WT *Bcl3* promoter, supporting the role of SP1 as a transcriptional activator (Supplementary Fig. S10D). Interestingly, we also observed non-specific effects of SP1 on transcriptional activation, as evidenced by increased luciferase activity in constructs containing G6A, G6/8A, and GI/IVA promoters. Consistent findings were obtained from qPCR analysis, where SP1 overexpression elevated *Bcl3* transcript levels, while SP1 knockdown significantly reduced these levels (Supplementary Figs S2D and S10E). Based on these observations, we hypothesized that G4-dependent SP1 condensation promotes transcription. To test this, we employed a transcription reporter system using a CFP-labeled PP7-coat protein (PCP-CFP) and PP7-containing reporter plasmids [77]. We inserted twelve PP7 RNA hairpin sequences (12 × PP7) into the 3′ region of the pGL4.11-Bcl3-WT or mutant luciferase reporter plasmids, generating pGL4.11-Bcl3-12 × PP7 constructs (Fig. 4A). The PP7 hairpins form RNA stem-loop structures that are specifically recognized by PCP-CFP, enabling visualization of transcriptional activity via CFP foci. To assess the relationship between SP1 condensation and transcription, we co-transfected MDA-MB-231 cells with SP1-mCherry, PCP-CFP, and the Bcl3-12 × PP7 reporter plasmids and analyzed the cells using confocal microscopy. Transfection with the reporter plasmid alone led to enlarged SP1 foci, suggesting recruitment of SP1 to the plasmid irrespective of exogenous SP1 expression (Fig. 4B and Supplementary Fig. S10F). When all components were co-transfected, mCherry and CFP foci co-localized in the nucleus (Fig. 4C), indicating SP1 condensates overlapped with active transcription sites. In a time-lapse analysis, cells were first transfected with SP1-mCherry and the reporter plasmid, followed by PCP-CFP transfection the next day. An increase in CFP signal was observed within preformed SP1 condensates over time (Supplementary Fig. S10G and H), supporting a positive link between SP1 condensation and transcriptional activation in cells.

To validate the role of G4 formation within the *Bcl3* promoter in SP1 condensation and transcriptional activation, we compared SP1 condensation and transcriptional output using WT and MT (G6/8A or GI/IVA) *Bcl3* promoter constructs. Co-transfection with the WT *Bcl3* promoter resulted in significantly higher levels of both SP1 condensates and CFP foci compared to the MT promoters (Fig. 4D and E), demonstrating that G4 structures facilitate SP1 condensation and promote transcription. The involvement of G4 in this process was further supported by experiments using the G4-binding ligands TMPyP4 and PDS. Cells co-transfected with SP1-mCherry, PCP-CFP, and pGL4.11-Bcl3-12 × PP7 and treated with TMPyP4 exhibited reduced formation of both SP1 condensates and CFP foci, whereas PDS treatment significantly increased both features (Supplementary Fig. S11A and B). Genomic analyses of publicly available ChIP-seq data revealed that SP1 co-localizes with MED1 and RNA Polymerase II (Pol II) at the *Bcl3* promoter (Fig. 1C and Supplementary Fig. S1B). Given that both MED1 and Pol II are known components of TCs [78, 79], we examined their spatial relationship with SP1. Confocal microscopy showed that overexpressed MED1-IDR and endogenous Pol II co-localized with endogenous SP1 condensates (Fig. 4F and G), indicating that SP1 recruits these factors to form transcriptionally active condensates. Collectively, these results demonstrate that G4 structures in the *Bcl3* promoter drive transcriptional activation by promoting SP1 condensation, which in turn recruits MED1 and Pol II to assemble TCs.

Previous studies have reported that SP1 exhibits synergistic transcriptional activity when multiple SP1 binding sites are present [33]. To examine whether this synergy is linked to SP1 condensation, we performed luciferase reporter assays using plasmids containing either a tandem duplication of the WT Bcl3 promoter (pGL4.11-WT2), a combination of WT and G6/8A promoters (pGL4.11-WT-G6/8A), or a tandem duplication of the G6/8A promoter (pGL4.11-G6/8A2) (Supplementary Fig. S11C). We first evaluated the effect of these constructs on endogenous SP1 condensation via immunofluorescence. Transfection with pGL4.11-WT_2_ did not significantly alter SP1 condensate area compared to pGL4.11-WT (99% relative area; Supplementary Fig. S11D–F). In contrast, pGL4.11-WT-G6/8A and pGL4.11-G6/8A2 increased SP1 condensate area to 141% and 124%, respectively, suggesting enhanced recruitment of SP1 despite reduced G4 content. Luciferase reporter assays showed that pGL4.11-WT2 retained 99% of the transcriptional activity observed with pGL4.11-WT, whereas pGL4.11-G6/8A, pGL4.11-WT-G6/8A, and pGL4.11-G6/8A2 drove 52%, 225%, and 206% of WT activity, respectively (Supplementary Fig. S11G). These results indicate that the combination or duplication of WT and mutant promoter elements enhances SP1-driven transcription, potentially through cooperative interactions independent of G4-mediated condensation. *In vitro* condensation assays revealed that duplication of the WT promoter resulted in SP1 condensation levels at approximately 80% of those observed with the single WT promoter (Supplementary Fig. S11H and I). In contrast, the combination of WT and G6/8A promoters or the duplication of the G6/8A promoter led to 2.0- and 1.5-fold increases in SP1 condensation, respectively—findings consistent with our cell-based experiments. Taken together with previous studies showing that SP1 self-association is essential for its synergistic activity [31], these results suggest that SP1 condensation may be a key mechanistic driver of SP1-mediated transcriptional synergism.

### Intrinsically disordered domain, transactivation domains, and DNA binding domain are crucial for SP1 condensation and SP1 activity

**Figure 5.**
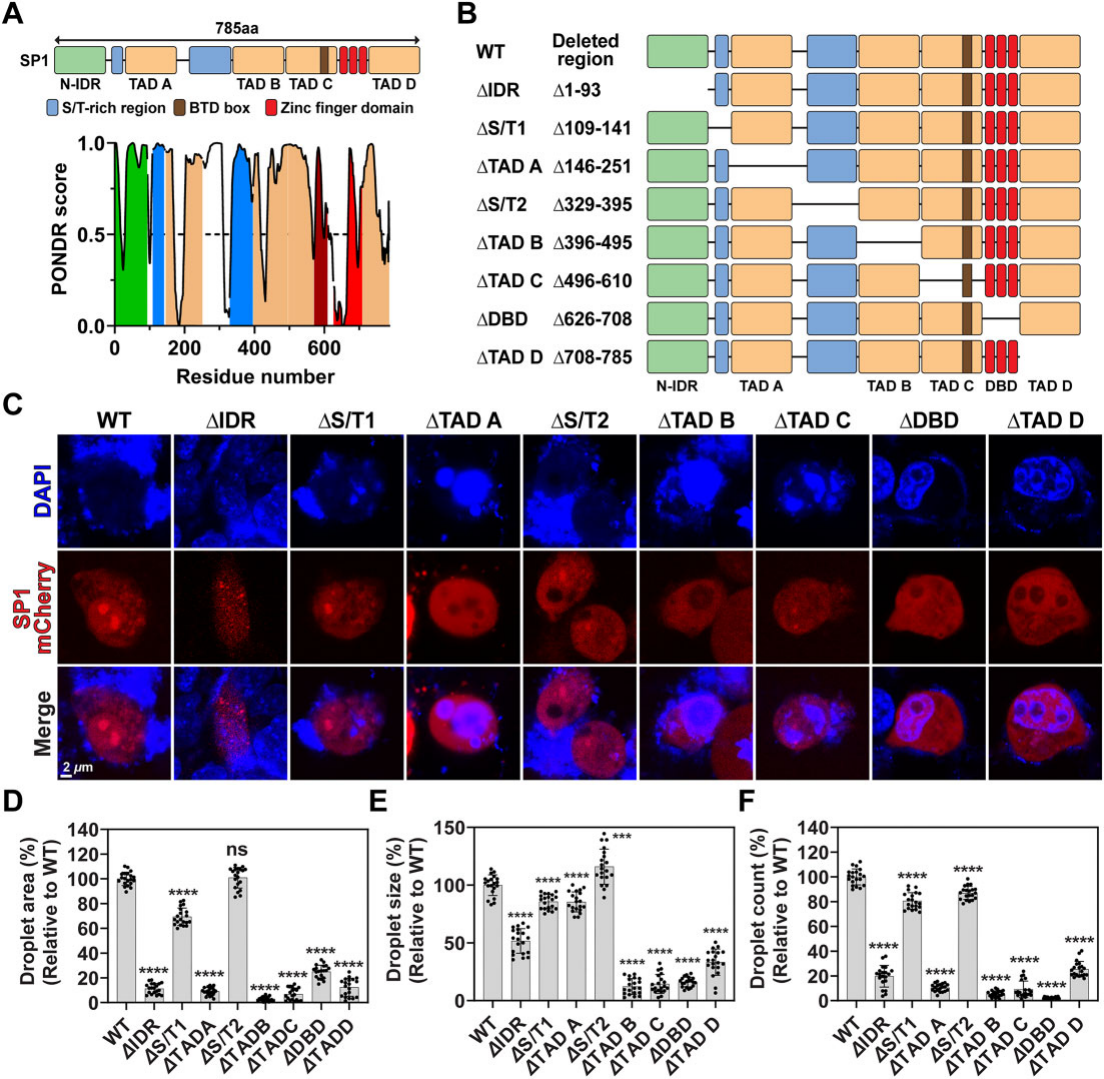
TADs and DBD of SP1 are crucial for SP1 condensation. (**A**) Schematic representation of the full-length human SP1 protein (UniProt ID: P08047) (top) and the intrinsic disorder prediction across the amino acid sequence, as determined by PONDR (Predictor of Natural Disordered Regions) software (bottom). (**B**) Schematic diagrams of WT and domain-deletion mutants (MTs) of SP1. The deleted regions in each mutant construct are indicated by amino acid residue numbers. (**C–F**) Representative FS images of MDA-MB-231 cells expressing WT or mutant SP1-mCherry constructs. (**C**) Images were captured at 40× magnification; nuclei were counterstained with DAPI. Enlarged single-cell images are shown. Quantification of total condensate area (**D**), average droplet size (**E**), and droplet count (**F**), each normalized to values from WT SP1-mCherry-expressing cells. Graphs represent mean ± standard deviation from ten randomly selected fields. Experiments were independently performed twice.

SP1 is a modular protein composed of an *N*-terminal intrinsically disordered region (N-IDR), two serine/threonine-rich regions (S/T1 and S/T2), a DNA-binding domain (DBD), and four transactivation domains (TAD-A, B, C, and D) (Fig. 5A). Except for the DBD, most regions are predicted to be intrinsically disordered, indicating that SP1 is largely disordered in nature (Fig. 5A). Previous studies have shown that each TAD contributes differently to transcriptional activation; TAD-A, B, and D are essential for full activity, whereas TAD-C plays a less prominent role [33]. To determine the role of each SP1 domain in condensation and transcriptional regulation, we performed luciferase reporter assays in MDA-MB-231 cells co-transfected with the pGL4.11-Bcl3 reporter and plasmids expressing either WT SP1 or domain-deletion mutants (Fig. 5B). Expression levels varied among mutants: *Δ*TAD-B and *Δ*TAD-C were expressed at levels comparable to WT, while *Δ*IDR, *Δ*S/T1, *Δ*TAD-A, and *Δ*S/T2 were expressed at 1.5–2-fold higher levels. In contrast, *Δ*DBD and *Δ*TAD-D showed reduced expression (∼0.6-fold of WT) (Supplementary Fig. S12A and B). Luciferase activity was generally reduced in most SP1 mutants compared to WT. However, *Δ*S/T1, *Δ*TAD-A, and *Δ*S/T2 exhibited higher or comparable transcriptional activity (150%, 91%, and 230% of WT, respectively), likely due in part to their elevated expression levels (Supplementary Fig. S12C). Despite a 1.5-fold increase in protein level, *Δ*IDR showed only 50% of WT activity, underscoring its importance. Similarly, *Δ*TAD-B and *Δ*TAD-C displayed 58% and 29% of WT activity, respectively, despite similar expression levels. To further assess how SP1 domains influence condensation and transcription, we generated domain-deletion mutants of SP1 fused to mCherry and visualized condensate formation by confocal microscopy. WT SP1, *Δ*S/T1, and *Δ*S/T2 formed distinct nuclear condensates, whereas *Δ*IDR, *Δ*TADs, and *Δ*DBD mutants were diffusely distributed, suggesting that the IDR, DBD, and TADs are critical for condensation (Fig. 5C). These observations closely parallel the luciferase assay results, highlighting a strong correlation between SP1’s condensation ability and its transcriptional activity. To explore domain-specific responses to G4 and dsDNA, we compared SP1 condensation using reporter plasmids containing either the WT or GI/IVA *Bcl3* promoter. Most domain-deletion mutants were unable to form condensates with either promoter (Supplementary Fig. S12D). Interestingly, deletion of the S/T2 domain did not disrupt SP1 condensation with either promoter. Although *Δ*S/T1 and *Δ*S/T2 mutants showed similar expression levels and condensation with the WT promoter, only *Δ*S/T2 could induce condensation with the GI/IVA promoter, suggesting a potential inhibitory role of the S/T2 domain in dsDNA-mediated SP1 condensation. To validate these findings functionally, we performed PCP–PP7 reporter assays in MDA-MB-231 cells expressing SP1 WT, *Δ*IDR, *Δ*S/T2, *Δ*DBD, or *Δ*TAD-D. Cells expressing *Δ*S/T2 exhibited enhanced CFP foci formation, with a 126% increase in CFP droplet area and a 186% increase in droplet count relative to SP1 WT (Supplementary Fig. S12E–H). Conversely, CFP foci were markedly reduced in cells expressing *Δ*IDR, *Δ*DBD, or *Δ*TAD-D. Taken together, these results demonstrate that the IDR, four TADs, and DBD are essential for SP1 condensation and transcriptional function. In contrast, the S/T2 domain appears to play a regulatory—possibly inhibitory—role, while deletion of the S/T1 domain had minimal impact on SP1 activity or condensation.

### RNA G4 regulates SP1-dependent transcription by modulating SP1 condensation

**Figure 6.**
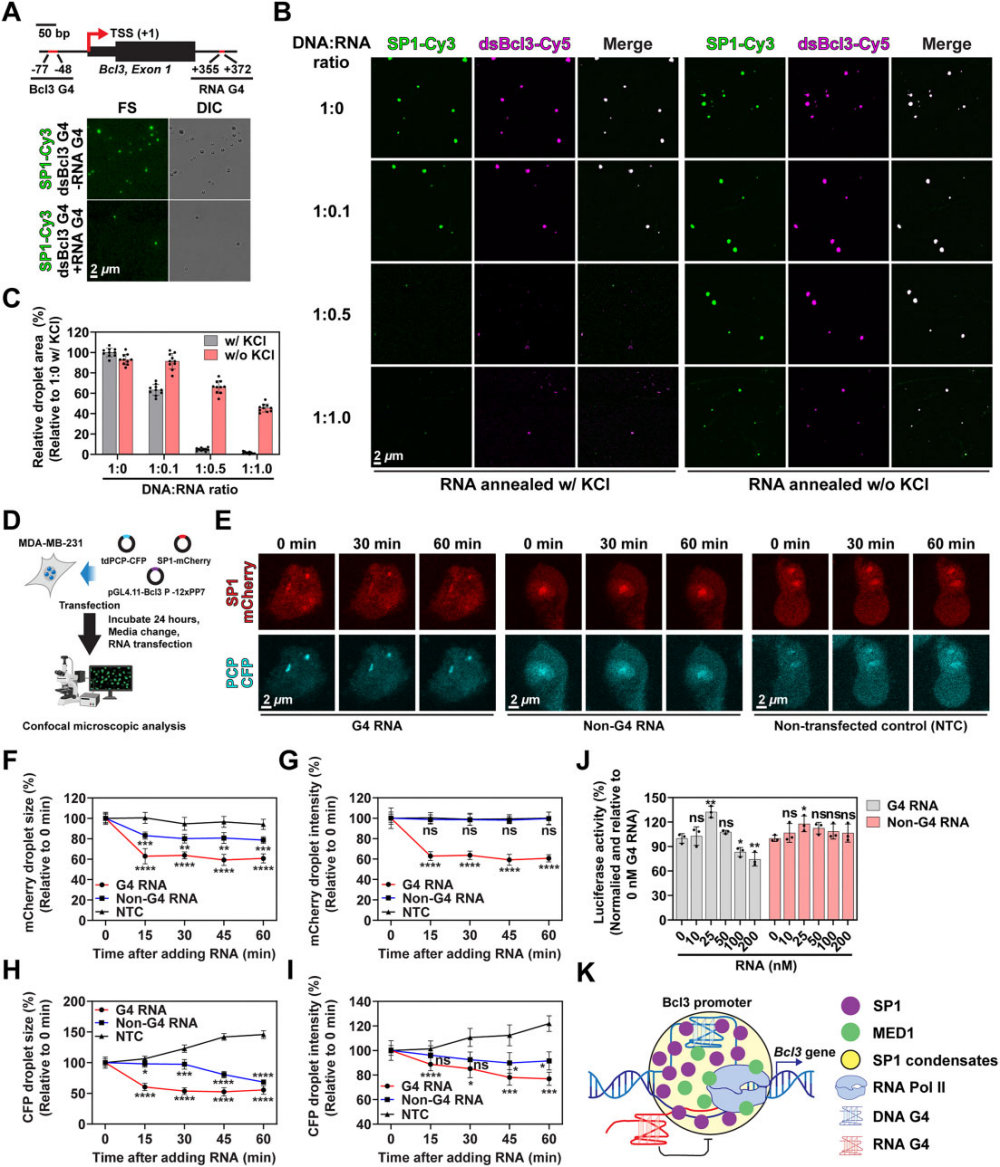
RNA influences SP1-mediated transcriptional regulation by modulating SP1 condensation. (**A**) Schematic of the *Bcl3* gene locus near the TSS (top). The genomic positions of the G4-forming sequences in the promoter (dsBcl3 G4) and in the intronic region (RNA G4) are indicated. *In vitro* condensation assay of SP1-Cy3 with dsBcl3 G4 in the presence or absence of RNA G4 is shown below. (**B**) *In vitro* condensation assay of SP1-Cy3 and dsBcl3 G4-Cy5 in the presence of RNA G4 annealed with or without KCl across various DNA:RNA ratios. (**C**) Quantification of SP1-Cy3 condensate area relative to the condition with DNA:RNA ratio of 1:0 (with KCl). Data represent mean ± standard deviation from ten randomly selected regions. Experiments were independently performed twice. (**D**) Schematic of time-lapse confocal imaging using the PP7–PCP transcription reporter system following RNA transfection. (**E**) Representative FS images of MDA-MB-231 cells co-transfected with SP1-mCherry, tdPCP-CFP, and the pGL4.11-*Bcl3*-12 × PP7 reporter plasmid. After 24 h, media were replaced and cells were transfected with 100 nM G4 RNA or non-G4 RNA. Images were acquired at 40× magnification; close-up views of individual cells are shown. (**F–I**) Quantification of droplet size and fluorescence intensity over time for SP1-mCherry (**F, G**) and PCP-CFP (**H, I**) condensates following transfection with G4 RNA, non-G4 RNA, or no RNA (NTC). Values are shown relative to those at 0 min. Data represent mean ± standard deviation from ten nuclei. Experiments were performed independently twice. (**J**) Luciferase assay using the pGL4.11-WT reporter plasmid in MDA-MB-231 cells co-transfected with varying concentrations of G4 RNA or non-G4 RNA. Luciferase activity was normalized to Renilla luciferase from co-transfected pRL-TK and expressed relative to non-transfection control. Data represent mean ± standard deviation from at least three independent experiments. (**K**) Model of SP1 condensation-dependent transcriptional regulation of the *Bcl3* gene. Promoter G4 structures facilitate SP1 condensation, which recruits transcriptional coactivators such as MED1 and RNA Pol II to form TCs. Intronic RNA G4 enhances SP1 condensation at low concentrations and dissolves it at high concentrations, providing a positive and negative feedback loop for *Bcl3* transcription.

RNAs are known components of TCs and play important roles in regulating transcription [80, 81]. Given that G4-forming RNAs are particularly implicated in transcriptional control [82], we investigated the effects of RNA and RNA G4 structures on SP1-mediated transcriptional condensation. We identified a putative G4-forming RNA upstream of *Bcl3* exon 1 (chr19:45 252 319–45 252 336; 18 nt) with a high QGRS score of 40, although it lacks an SP1 binding site (Fig. 6A, top; Supplementary Table S1). Using EMSA, we confirmed SP1 binding to the RNA both with and without G4-promoting conditions (KCl) (Supplementary Fig. S13A and B). However, SP1 condensation was more efficiently induced by RNA under G4-forming conditions (RNA (KCl)) than under non-G4 conditions (RNA (No KCl)) (Supplementary Fig. S13C and D), although this effect was weaker than that induced by dsBcl3 G4 (Supplementary Fig. S13E and F). Notably, RNA (KCl) inhibited SP1 condensation when co-incubated with dsBcl3 at equal concentrations, as evidenced by a reduction in Cy3-labeled foci and phase-separated droplets (Fig. 6A, bottom). Increasing concentrations of RNA—especially G4-forming RNA—further suppressed SP1 condensation (Fig. 6B). We also assessed whether RNA could dissolve preformed SP1 condensates by adding RNA to existing droplets and monitoring dissolution over time using confocal microscopy (Supplementary Fig. S13G). Controls included dsDNA (Bcl3 WT and G6/8A) and G4-deficient RNA (G-to-A mutant). Both RNA and dsDNA dissolved SP1 condensates, with dissolution significantly enhanced by G4 formation (Supplementary Fig. S13H and I). To confirm these effects in cells, we transfected MDA-MB-231 cells with either G4 or non-G4 RNA. Immunofluorescence analysis showed a dose-dependent decrease in SP1 condensate area and size upon RNA transfection, with G4 RNA having a stronger effect (Supplementary Fig. S14A–D). We next assessed the transcriptional consequences by co-transfecting G4 or non-G4 RNA along with PCP-CFP, pGL4.11-Bcl3-12 × PP7, and SP1-mCherry plasmids. One day after DNA transfection, RNA was introduced (Fig. 6D). G4 RNA significantly reduced the size and intensity of SP1-mCherry droplets (Fig. 6E–G, red line), whereas non-G4 RNA had a milder effect (blue line). Similarly, G4 RNA strongly suppressed PCP-CFP droplet formation, indicating reduced transcriptional activity (Fig. 6H, I, red line), while non-G4 RNA had modest effects (blue line). Consistent with these findings, luciferase reporter assays showed a marked reduction in transcriptional activity at G4 RNA concentrations above 25 nM, with non-G4 RNA showing only minimal suppression (Fig. 6J). To further evaluate whether co-transcriptionally generated G4 RNA interferes with SP1 condensation and transcriptional activity, we constructed reporter plasmids containing a 201-bp G-rich region from the *Bcl3* gene (chr19:45 252 219–45 252 419), which is predicted to form a G4 structure in the transcript. This region was cloned downstream of either the WT *Bcl3* promoter or the G6/8A mutant promoter (Supplementary Fig. S14E). CD spectroscopy confirmed that the 18-nt G-rich sequence within the *Bcl3* transcript adopted a G4 structure in the WT construct but not in the mutant (Supplementary Fig. S14F). Using an *in vitro* transcription assay with HeLa nuclear extract, we detected a transcript of approximately 500 nt, corresponding to the combined length of the 12 × PP7 cassette (312 nt) and the downstream G-rich region (201 bp) (Supplementary Fig. S14G). Transcriptional output was higher when the G4-forming sequence was disrupted in the transcript (mutant), indicating that the G4 structure in the RNA may act as a negative regulator of transcription (Supplementary Fig. S14G and H). SP1 supplementation increased overall transcript levels for both WT and mutant promoters, with transcription from the WT promoter remaining higher than from the G6/8A mutant under SP1-stimulated conditions. To assess this phenomenon in a cellular context, we performed live-cell imaging using the PP7–PCP reporter system. Cells were co-transfected with plasmids expressing SP1-mCherry, PCP-CFP, and a PP7-tagged *Bcl3* reporter construct containing either the WT or mutant G4 region. CFP signals, representing nascent transcripts, appeared more prominent when the transcript lacked the G4 motif (Supplementary Fig. S14I and J), consistent with enhanced transcription in the absence of the G4 structure. Collectively, these findings suggest that G4 RNA can disrupt SP1 condensation and interfere with transcription, likely through a mechanism facilitated by G4 formation within the transcript.

## Discussion

TCs provide a framework to explain the local enrichment of transcriptional machinery at active genomic loci [83]. In this model, TFs and coactivators undergo phase separation at promoters and enhancers, facilitating the recruitment of RNA polymerase to drive transcription [84, 85]. In this study, we investigated the role of G4 structures in modulating the condensation of SP1, a G4-binding TF, and its regulatory impact on *Bcl3* gene transcription. Based on our findings, we propose the following model for G4-dependent transcriptional regulation of the *Bcl3* gene (Fig. 6K): G4 formation within the *Bcl3* promoter promotes the recruitment of SP1 and facilitates its condensation. This SP1 condensation, in turn, promotes co-condensation with the coactivator MED1 and RNA Polymerase II, establishing a TC at the promoter. Furthermore, nascent RNA transcripts modulate this process by regulating SP1 condensation in an RNA concentration-dependent manner, adding an additional layer of control to transcriptional output.

TF condensation has been shown to be regulated by local chromatin architecture and motif features, such as motif density, binding affinity, and epigenetic status [13, 86]. In this study, we demonstrated that G4 structures enhance SP1 binding affinity to DNA, thereby promoting SP1 condensation. While G4s can form under low-salt conditions, optimal SP1 condensation required higher ionic strength, consistent with prior findings that phase separation is modulated by ionic conditions and macromolecular crowding [17, 72, 73]. Given that SP1 has been reported to self-associate in a DNA-dependent manner, we propose that initial binding of SP1 to a G4 structure facilitates the recruitment of additional SP1 molecules through cooperative interactions [31]. Accordingly, the kinetic parameters obtained from our BLI experiments should be interpreted with caution, as the SP1–G4 interaction does not conform to a simple 1:1 binding model. The dissociation constant (*K*_D_) for SP1 binding to G4 DNA measured by BLI (2.36 nM) was significantly lower than that obtained by EMSA (19.1 nM) or previously reported values from EMSA and fluorescence correlation spectroscopy (23–32 nM) [66, 67]. This discrepancy likely reflects contributions from both SP1–DNA interactions and SP1 self-association. Moreover, SP1 dissociated more rapidly from non-G4 DNA than from G4-containing DNA (Supplementary Fig. S6B), while association rates remained comparable. These findings suggest that G4 structures extend SP1 residence time on DNA, thereby stabilizing condensation. EMSA results further supported this model, showing that SP1 binds cooperatively to G4 DNA with a high Hill coefficient (4.42), in contrast to non-G4 DNA (0.87) (Fig. 2C). This cooperative behavior may enable SP1 to form condensates even at low G4 concentrations in cells, where G4 structures are sparsely distributed across chromosomes. The effect of G4 on SP1 binding kinetics may also explain the heterogeneous binding dynamics observed for SP1 at genomic targets [87]. Future single-molecule studies will be essential to elucidate how G4 modulates SP1 kinetics and its role in TC formation.

TCs are associated with robust transcriptional activity by facilitating the recruitment of coactivators and RNA polymerase II (Pol II) [11, 88], organizing local chromatin environments [16, 86], and modulating coactivator function [89]. Our findings demonstrate that G4-dependent SP1 condensation promotes transcription, supporting a regulatory role for G4 structures in SP1-mediated gene activation. SP1 is known to interact with the *C*-terminal domain of the TATA-box binding protein (TBP) via its TAD-A and TAD-B domains, promoting assembly of the pre-initiation complex (PIC) [90, 91]. Consistent with this function, we observed that SP1 condensates co-localize with MED1 and RNA Pol II in cells—paralleling previous observations of G4-dependent MAZ condensates enriched for coactivators and Pol II [17]. These results suggest that SP1 condensates may contribute to both PIC assembly and maintenance of active transcriptional hubs. Our analysis of publicly available ChIP-seq data for additional G4-binding TFs, including MAZ, NRF1, and E2F4, suggests that G4 structures may influence the selective recruitment or exclusion of these factors into TCs [84]. These findings underscore the complex and context-dependent role of G4 elements in shaping the molecular composition of nuclear condensates.

Consistent with previous reports of SP1’s synergistic activity in transcription [32, 33], we observed a positive correlation between promoter activity and SP1 condensation in artificially combined Bcl3 promoters. These findings suggest that SP1 condensation may serve as a mechanism to enhance SP1-driven transcriptional synergy. In our assays, combining the G6/8A promoter with either itself or the WT promoter resulted in a 4-fold and 5-fold increase in promoter activity and SP1 condensation, respectively, compared to the single G6/8A construct (Supplementary Fig. S11C–G). In contrast, no synergistic effect was observed when two WT promoters were combined. A recent study suggests that optimal transcriptional regulation can arise from the arrangement of strong and weak TF binding motifs [92]. Based on this, we propose that heterotypic motif pairing—specifically the combination of a strong (WT) and weak (G6/8A) SP1 motif—promotes cooperative SP1 binding and condensation. In this model, the WT motif functions as a high-affinity anchor, while the adjacent G6/8A motif serves as a secondary binding site that stabilizes multivalent interactions without competing for SP1 occupancy. This configuration may enhance cooperative binding and support the assembly of SP1 condensates [92]. Although the mechanism underlying the reduced SP1 condensation and transcription observed with tandem WT promoters remains unclear, our *in vitro* data suggest that intra-array competition among high-affinity motifs may disrupt optimal spacing or stoichiometry, thereby impeding condensate formation. Future studies will be essential to define the spatial and quantitative parameters of SP1–DNA interactions that drive TC assembly.

In this study, we investigated the contribution of specific SP1 domains to its transcriptional activity and condensate formation. Previous studies have demonstrated that SP1 multimerization is essential for DNA looping and transcriptional synergism [31–33]. Consistent with this, we found that deletion of SP1 TADs—which mediate multimerization and looping [33, 93]—abolished both condensate formation and transcriptional activation (Fig. 5 and Supplementary Fig. S12). Deletion of the DBD similarly abrogated SP1 condensation and transcriptional output, reinforcing the idea that SP1 condensation is DNA-dependent. Interestingly, the *Δ*S/T2 mutant retained robust condensation activity on both G4 and non-G4 DNA, whereas WT SP1 and all other domain-deletion mutants failed to form condensates under these conditions. These results suggest that the S/T2 domain may act as a negative regulatory element that suppresses SP1 condensation in the absence of G4 structures. Supporting this hypothesis, the S/T2 region is a known site of post-translational modifications—such as MAPK/ERK-mediated phosphorylation at Thr355—which regulate SP1’s activity, stability, and protein–protein interactions [94]. Although speculative, our findings imply that the S/T2 domain plays a key role in tuning SP1 condensation dynamics.

RNA plays a key role in transcriptional regulation by modulating biomolecular condensation [80, 95]. Our results show that RNA derived from the Bcl3 transcript can dissolve SP1 condensates both *in vitro* and *in vivo* (Fig. 6; Supplementary Fig. S13). Consistent with previous studies demonstrating a concentration-dependent effect of RNA on condensation [80], we observed that RNA promoted SP1 condensation at low concentrations, but dissolved condensates at higher concentrations (Supplementary Fig. S13C and D). A similar biphasic response was observed with G4 DNA: at low DNA concentrations (SP1:G4 = 1:0.1), robust SP1 condensate formation was observed, while increasing the G4 DNA concentration (SP1:G4 = 1:0.2) dramatically reduced condensation to ∼10% (Fig. 2G and H). These findings suggest that both G4 DNA and RNA function as biphasic modulators of SP1 condensation, capable of either promoting or disrupting condensates depending on their concentration. The dissolution of condensates by excess nucleic acids is likely due to disruption of local charge balance, a sequence-independent mechanism [80, 96]. However, we also found that nucleic acids capable of forming G4 structures more effectively disrupted SP1 condensates than their non-G4 counterparts. When G4 or non-G4 DNA/RNA was added to preformed SP1 condensates, G4-containing molecules more efficiently dissolved the condensates (Supplementary Fig. S13G–I). These results suggest that SP1 condensation is finely modulated by G-rich sequences with the capacity to form G4 structures. Together, these findings support a model in which SP1’s distinct binding affinities for RNA and DNA—modulated by the presence or absence of G4 structures—regulate SP1 condensation dynamics and, consequently, gene expression.

Although generally considered a ubiquitous TF, SP1 plays a crucial role in cell-type-specific gene expression programs and is frequently overexpressed in various cancers [19, 97–99]. Interestingly, SP1 also regulates its own expression, suggesting a complex self-regulatory mechanism that may amplify or fine-tune its influence within these gene networks (Supplementary Fig. S1A). Despite its biological significance, no specific inhibitors targeting SP1 have been developed to date, in part due to an incomplete understanding of its mechanistic regulation. In this study, we demonstrate that G4-dependent condensation represents a regulatory mechanism through which SP1 modulates the transcription of G4-associated genes. We demonstrated that G4-binding ligands, such as TMPyP4 and PDS, effectively modulate SP1 condensation. TMPyP4, a non-G4-selective ligand that binds G-rich sequences in double-stranded DNA, is known to induce conformational transitions and destabilize G4 structures [100, 101]. In contrast, PDS is a G4-selective ligand that specifically stabilizes G4 structures across diverse sequence and structural contexts [102]. These distinct properties were reflected in our experiments: TMPyP4 disrupted G4 structures within the *Bcl3* promoter and inhibited G4-dependent SP1 condensation (Supplementary Fig. S4A–D), while PDS stabilized the G4 structure and enhanced SP1 condensation. Notably, prior studies have shown that PDS derivatives can co-bind G4 structures with G4-binding proteins or the BG4 antibody [103, 104], supporting a model in which PDS facilitates TF recruitment. These findings provide a mechanistic explanation for how PDS enhances SP1-driven transcriptional activation at the Bcl3 promoter by stabilizing promoter G4 structures. While G4s have emerged as attractive therapeutic targets, efforts to modulate them using conventional small molecules—either to stabilize or destabilize G4 structures—have faced significant limitations, including poor specificity and context-dependent effects [105]. As an alternative strategy, directly targeting SP1 may offer a more precise and effective approach in cancer therapy. Our domain-deletion analysis revealed that all TADs and the DBD of SP1 are essential for G4-dependent SP1 condensation and transcriptional activity (Fig. 5; Supplementary Fig. S9). Notably, AlphaFold predicts that all TADs in SP1 are intrinsically disordered (AF-P08047-F1), highlighting the DBD as a structurally ordered region that could be exploited for rational drug design. Small molecules or screening platforms could be developed to specifically disrupt G4-dependent SP1 condensation by targeting the DBD, thereby attenuating SP1-mediated transcriptional activation. Although our current study focused on the regulation of the *Bcl3* gene in a breast cancer cell line, SP1 has been implicated in the regulation of several oncogenes—including *IGF1R*, *hTERT*, *TP53*, and *CDKN1A*—via G4 elements in their promoters [21–24]. Therefore, targeting SP1 offers a compelling therapeutic avenue with the potential to modulate oncogenic transcriptional networks across multiple cancer contexts. By intervening at the level of SP1 condensation, this strategy may enable more selective modulation of transcriptional programs critical for tumor progression.

## Supplementary Material

gkaf827_Supplemental_File

## Data Availability

Derived data supporting the findings of this study are available from the corresponding author (K.K.K.) on request.
