# Supplementary material for: G-quadruplex-dependent transcriptional regulation by molecular condensation in the Bcl3 promoter
Source: Nucleic Acids Res. 2025 Aug 30;53(16):gkaf827. doi: 10.1093/nar/gkaf827 (PMC12397910; doi:10.1093/nar/gkaf827)
Supplement: gkaf827_Supplemental_File [file gkaf827_supplemental_file.docx]

**Supplementary Figures and Tables**

**G-quadruplex-dependent transcriptional regulation by molecular condensation in the *Bcl3* promoter**

Wanki Yoo^1,†^, Yi Wei Song^1,†^_,_ Varun Bansal^1^, Kyeong Kyu Kim^1*^

^1^Department of Precision Medicine, Graduate School of Basic Medical Science (GSBMS), Institute for Antimicrobial Resistance Research and Therapeutics, Sungkyunkwan University School of Medicine, Suwon 16419, Republic of Korea

†The first two authors should be regarded as Joint First Authors.

*To whom all correspondence should be addressed: kyeongkyu@skku.edu

**
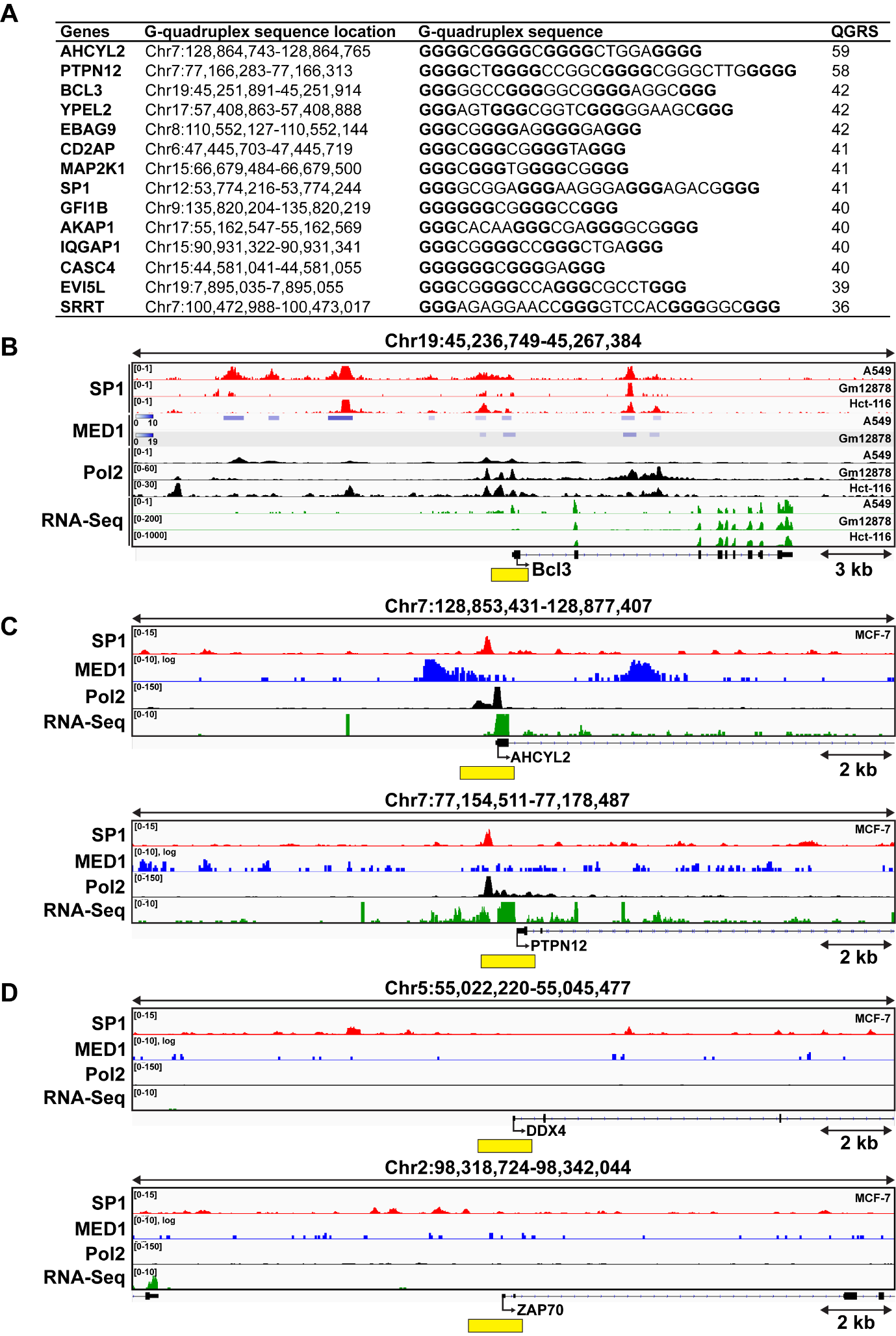
**

**Supplementary Figure S1. List of genes associated with SP1-enriched promoters in MCF-7 cells and ChIP-seq profiles of SP1, MED1, RNA polymerase II (Pol II), and RNA-seq data near the *Bcl3* gene and other loci across various cancer cell lines.** (**A**) List of genes associated with 14 SP1-enriched promoters. For each gene, genomic coordinates, the location and sequence of G-quadruplex (G4)-forming regions, and QGRS (Quadruplex-forming G-rich Sequences) scores calculated using the QGRS Mapper are summarized. **(B)** ChIP-seq profiles (raw reads) of SP1, MED1, and RNA Pol II, along with RNA-seq profiles (raw reads) near the *Bcl3* locus in lung adenocarcinoma (A549), lymphoblastoid (GM12878), and colorectal carcinoma (HCT-116) cell lines. Profiles were visualized using Integrated Genomics Viewer (IGV). **(C, D)** ChIP-seq profiles (raw reads) of SP1, MED1, and RNA Pol II, together with RNA-seq profiles (raw reads), near other SP1-enriched promoters (**C**) and non-SP1-enriched promoters (**D**) in MCF-7 cells. IGV was used for image generation.

**
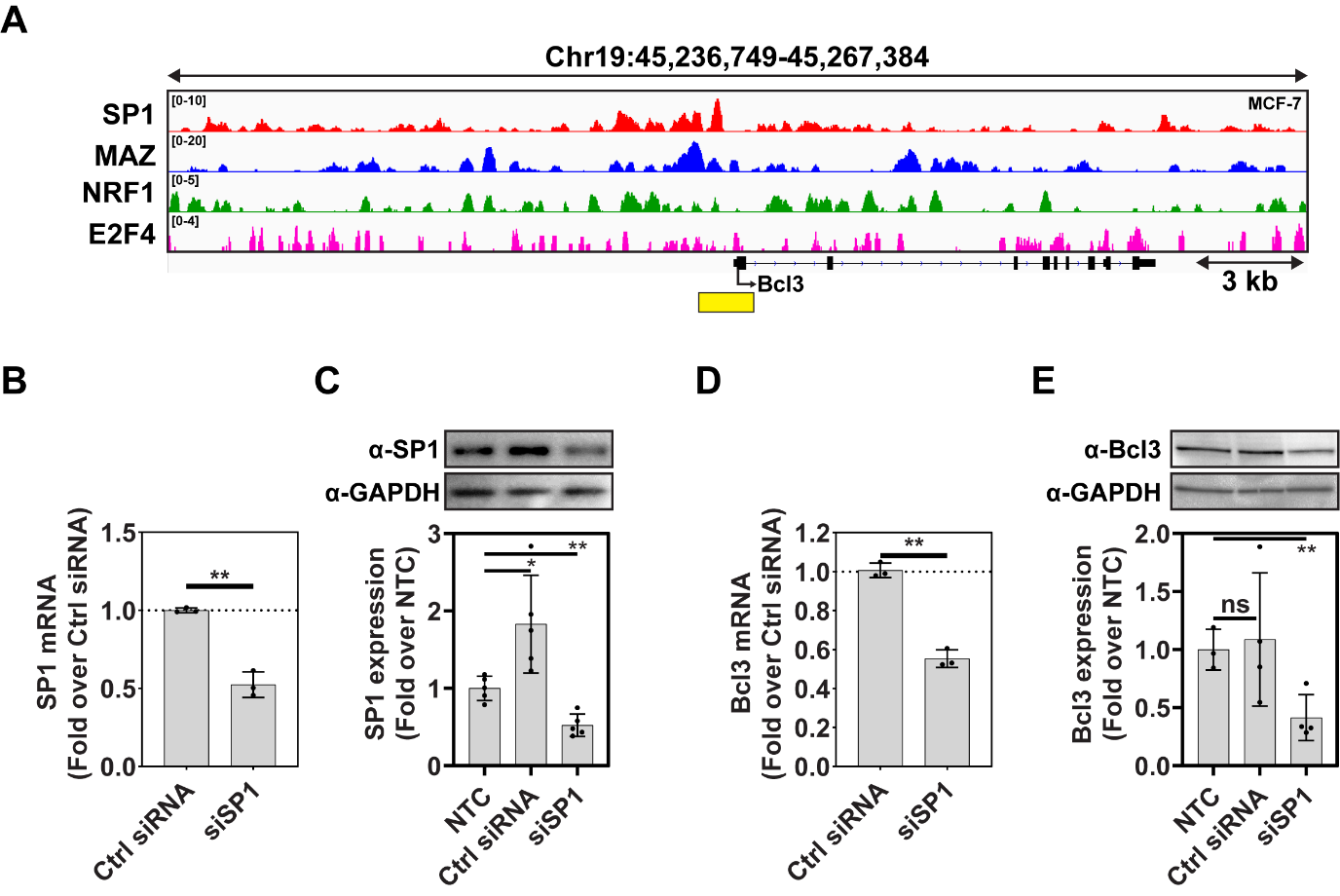
**

**Supplementary Figure S2. ChIP-seq profiles of G4-binding proteins at the *Bcl3* locus in MCF-7 cells, and gene knockdown analysis of SP1 and *Bcl3* in MDA-MB-231 cells. (A)** ChIP-seq profiles (raw reads) of SP1 (GEO: GSM2423902), MAZ (GEO: GSM2423262), NRF1 (GEO: GSE91522), and E2F4 (GEO: GSE105536) near the *Bcl3* gene in MCF-7 cells. The putative *Bcl3* promoter region is indicated by a yellow bar. Data were visualized using Integrated Genomics Viewer (IGV) software. **(B, C)** Quantitative RT-PCR (qRT-PCR) and Western blot analysis of SP1 expression following siRNA-mediated SP1 knockdown. **(D, E)** qRT-PCR and Western blot analysis of *Bcl3* expression following knockdown of SP1 or *Bcl3* in MDA-MB-231 cells.

**
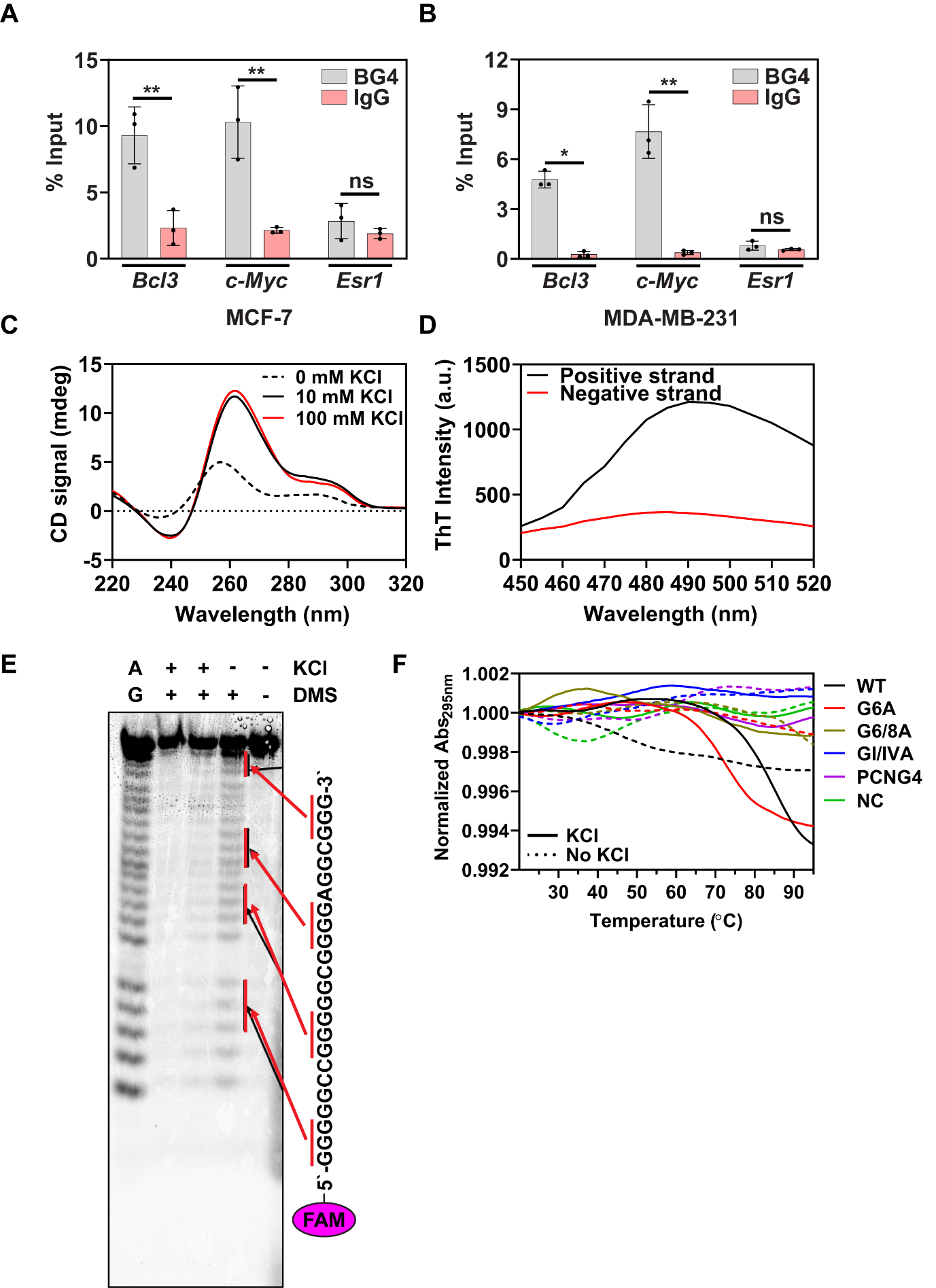
**

**Supplementary Figure S3. Validation of G-quadruplex (G4) formation in the *Bcl3* promoter using ChIP-qPCR, circular dichroism (CD), Thioflavin T (ThT) assay, dimethyl sulfate (DMS) footprinting, and UV melting analysis.** ChIP-qPCR using the BG4 antibody in MCF-7 (A) and MDA-MB-231 (B) cells. Enrichment at the *Bcl3*, *c-Myc*, and *Esr1* promoters was assessed. Normal IgG was used as a negative control. Values are shown relative to 5% input. Graphs display mean ± standard deviation from at least two independent experiments. **(C)** CD spectra of the *Bcl3* WT oligo annealed in 0, 10, or 100 mM KCl, showing K⁺-dependent G4 formation. **(D)** Thioflavin T (ThT) fluorescence assay of the *Bcl3* WT oligo (positive strand) and its complementary strand, annealed in 100 mM KCl. **(E)** DMS footprinting analysis of the *Bcl3* WT oligo. The 5′-FAM–labeled oligo was annealed in the presence or absence of 100 mM KCl and treated with 0.5% DMS. Reactions were quenched by adding 1 μg of calf thymus DNA. (**F**) UV absorption melting analysis of WT and MT G4 oligos. Absorbance at 295 nm was monitored as a function of temperature and normalized to the signal at 20 °C.


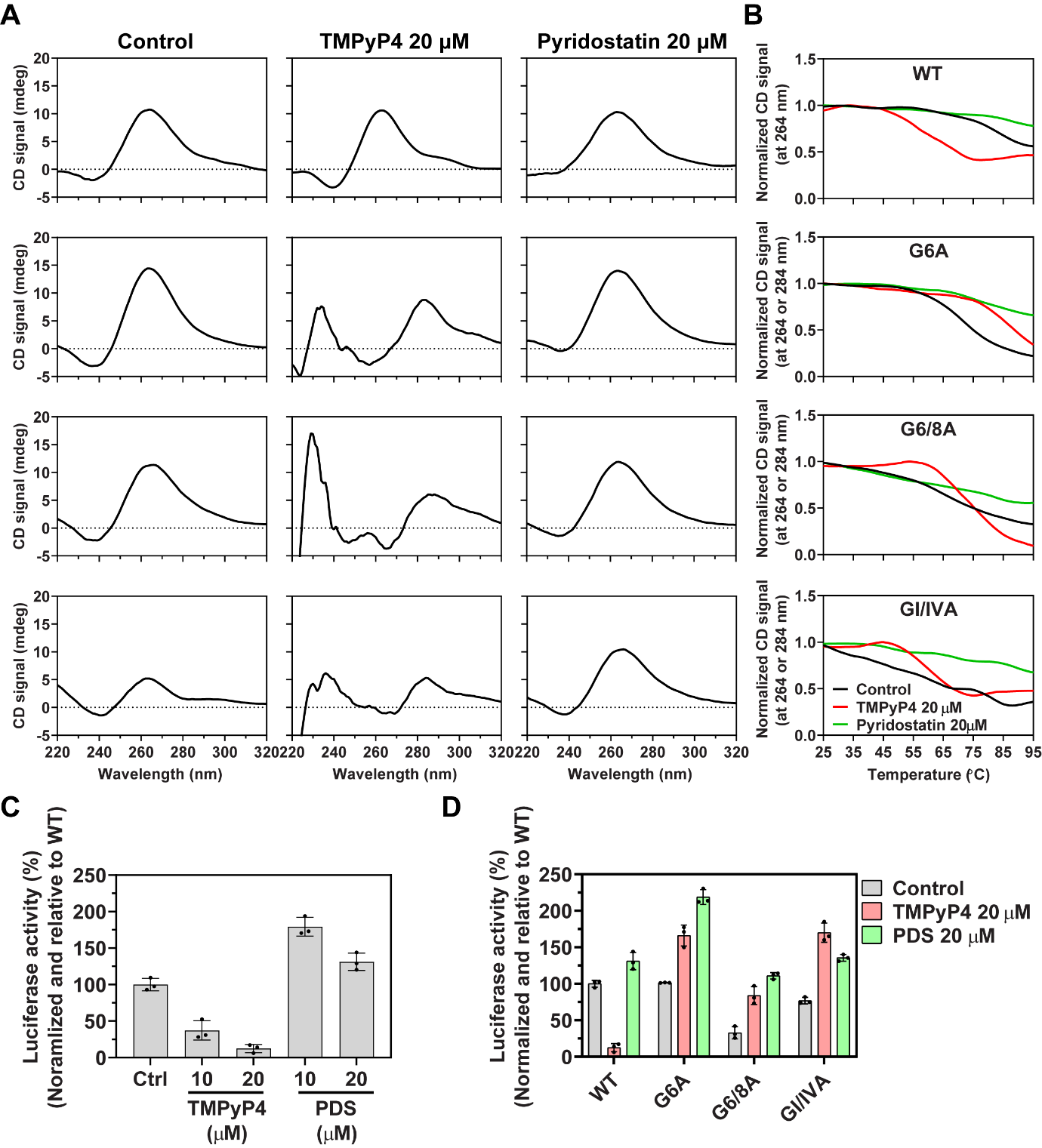


**Supplementary Figure S4. CD analysis and luciferase reporter assays of *Bcl3* WT and MT promoters in the presence of G4-stabilizing ligands.** (**A, B**) CD analysis of *Bcl3* WT and MT oligonucleotides treated with 20 μM TMPyP4 or Pyridostatin (PDS). (A) CD spectra of WT and MT oligos annealed in the presence of either ligand. (**B**) Melting temperature (Tm) analysis of the same samples, monitoring thermal stability from 25 °C to 95 °C. (**C**) Luciferase reporter assay using the pGL4.11-*Bcl3* WT promoter construct in MDA-MB-231 cells treated with 10 or 20 μM TMPyP4 or PDS. (**D**) Luciferase reporter assay using pGL4.11 constructs containing either WT or MT *Bcl3* promoter sequences in MDA-MB-231 cells treated with 10 μM TMPyP4 or PDS. All luciferase activities were normalized to Renilla luciferase activity from co-transfected pRL-TK plasmid and expressed relative to the untreated WT control. Graphs represent mean ± standard deviation from at least three independent experiments.


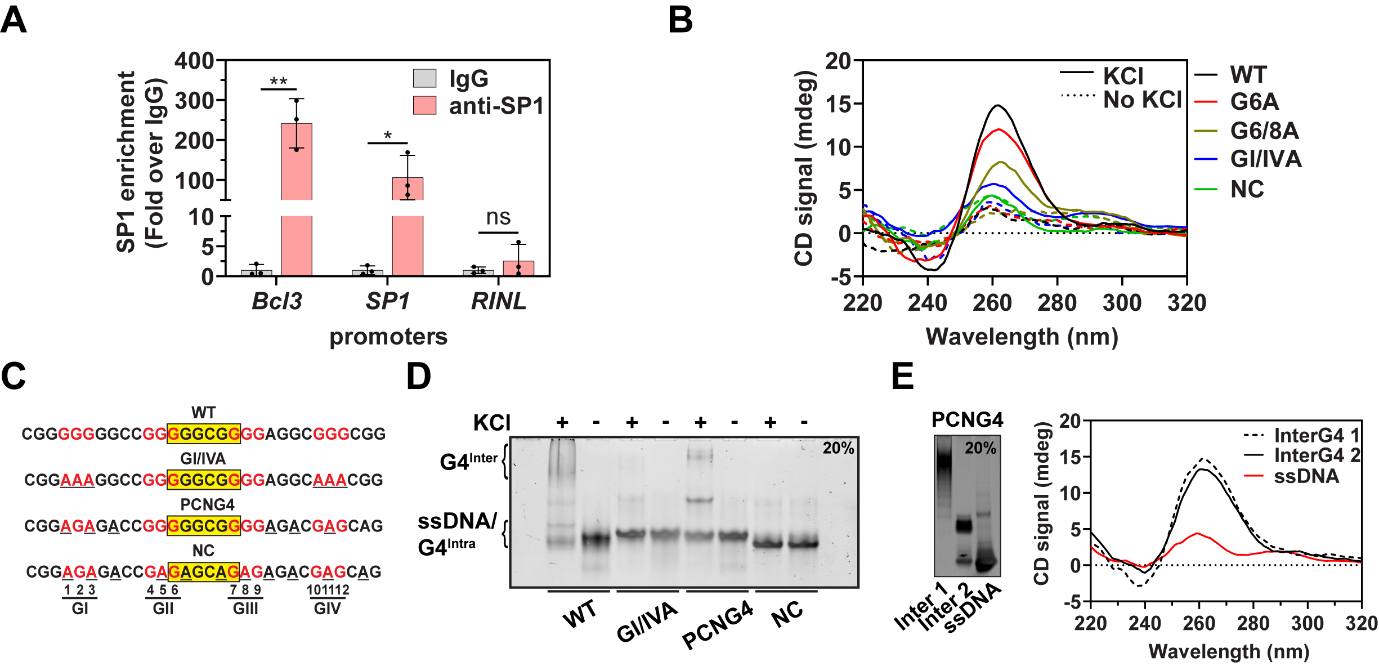


**Supplementary Figure S5. ChIP-qPCR and biophysical characterization of SP1 binding to *Bcl3* wild-type (WT) and mutant (MT) oligonucleotides.** (**A**) ChIP-qPCR analysis of SP1 binding at the *Bcl3*, *Sp1* (positive control), and *Rinl* (negative control) promoters in MDA-MB-231 cells using an anti-SP1 antibody. Normal IgG was used as a negative control. Enrichment is shown relative to IgG. Data represent mean ± standard deviation from at least two independent experiments. **(B)** CD spectra of 24-nt WT and mutant (*G6A*, *G6/8A*, *GI/IVA*, and negative control [NC]) oligonucleotides annealed in the presence (solid line) or absence (dashed line) of 100 mM KCl. **(C)** Sequences of WT and MT oligos used in electrophoretic mobility shift assays (EMSAs). **(D)** Native PAGE analysis (20%) of oligos annealed in the presence or absence of 100 mM KCl to assess G4 formation. Bands migrating more slowly than single-stranded DNA (ssDNA) were classified as intermolecular G4s (*G4*^inter^), while faster migrating bands were identified as intramolecular G4s (*G4*^intra^). (**E**) EMSA (left) and CD analysis (right) of PCNG4 species separated by size-exclusion chromatography. Samples were analyzed on a 20% native polyacrylamide gel. CD spectra were collected for each fraction to confirm G4 structure.


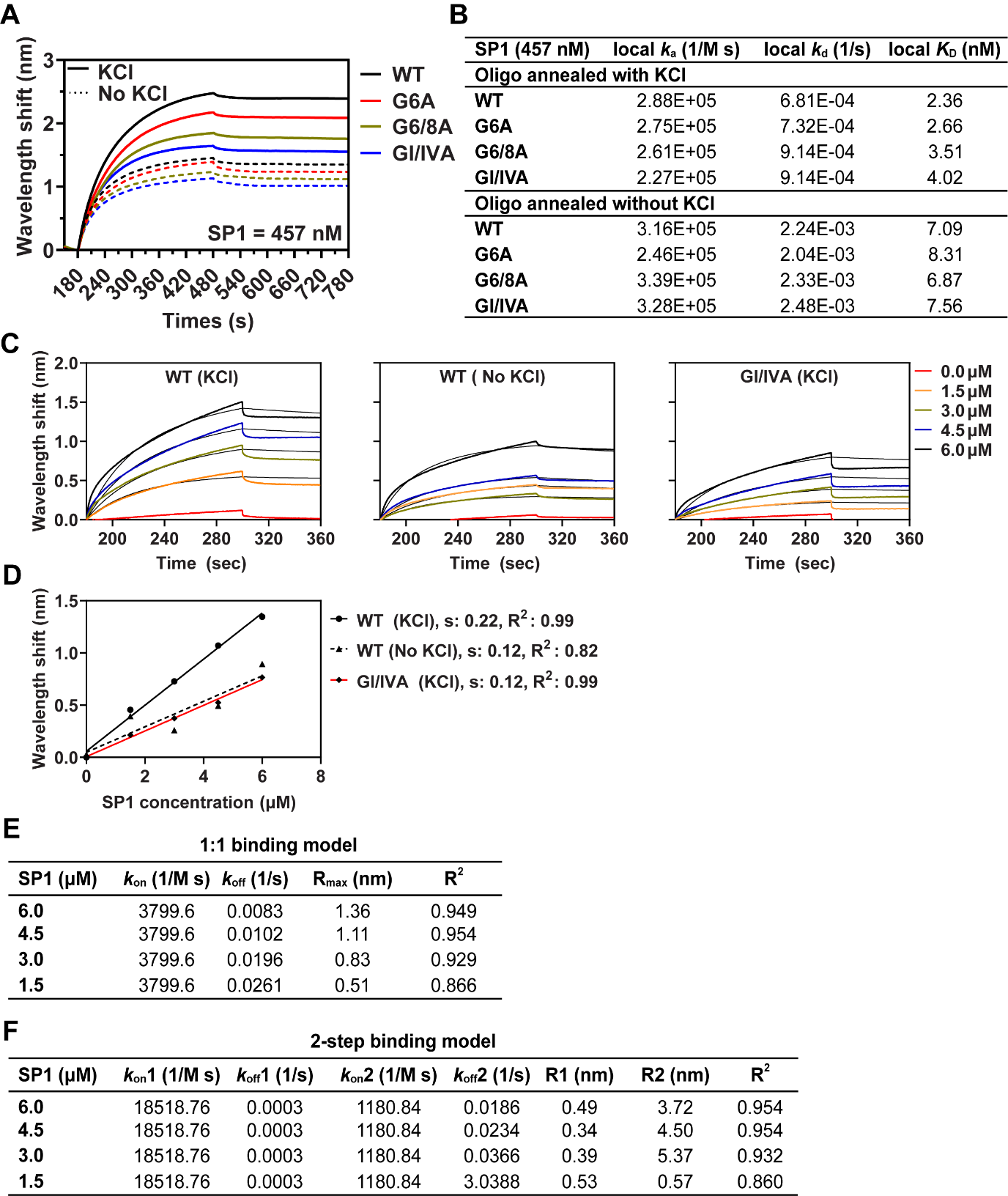


**Supplementary Figure S6. Bio-layer interferometry (BLI) and electrophoretic mobility shift assay (EMSA) analyses of SP1 binding to *Bcl3* WT and MT oligonucleotides. (A)** Representative BLI sensorgrams showing interactions between SP1 and oligonucleotides derived from the G4-forming region of the *Bcl3* promoter. Oligos were annealed in the presence (solid line) or absence (dashed line) of 100 mM KCl. (**B**) The local association rate (*k*_a_), dissociation rate (*k*_d_), and dissociation constant (*K*_D_) at 457 nM of SP1 were determined by locally fitting the curve to a 1:1 binding model. (**C**) BLI binding curves for 5′-biotin–labeled WT and GI/IVA oligonucleotides, annealed with or without 100 mM KCl. Oligos were immobilized on the sensor surface, and SP1 was applied at concentrations ranging from 0 to 6 μM. Association and dissociation phases were recorded. Curves were fitted to 1:1 global binding model. (**D**) Quantification of endpoint wavelength shifts from (C), plotted against SP1 concentration for WT and GI/IVA oligos. These data provide a comparative assessment of SP1 binding affinity. (**E-F)** Kinetic parameter derived from the 1:1 binding model and 2-step binding model. R_max_ represents the maximum equilibrium binding response. *k*_on_1 and *k*_off_1 are the association and dissociation rates of the first binding step, *k*_on_2 and *k*_off_2 are the association and dissociation rate of the second step in the 2-step binding model. R1 and R2 are the maximum equilibrium binding responses of the first and second steps, respectively.


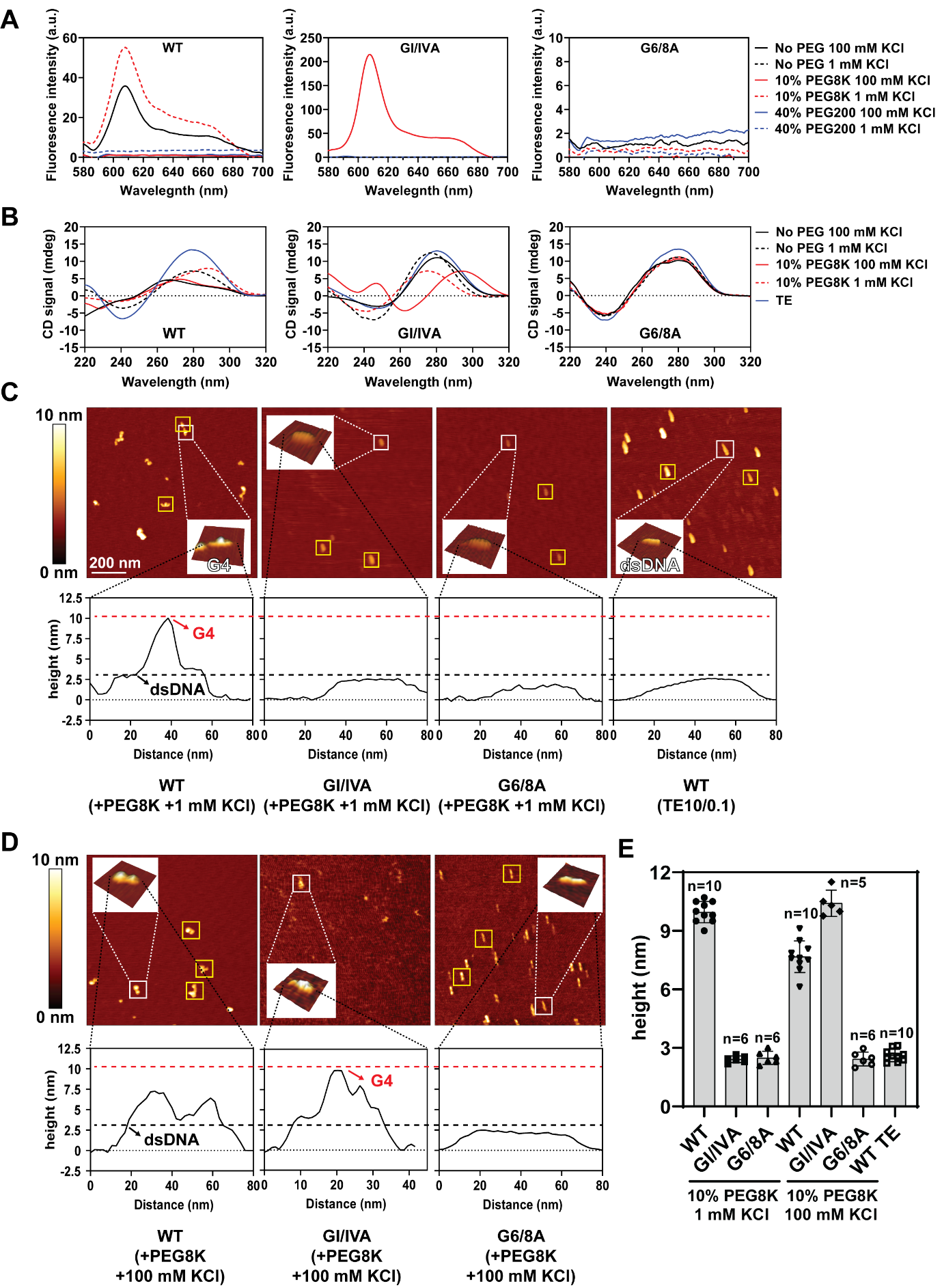


**Supplementary Figure S7. Analysis of G4 formation in double-stranded *Bcl3* promoter DNA (dsBcl3).** (**A**) N-methyl mesoporphyrin IX (NMM) fluorescence assay for 90-bp dsBcl3 WT, GI/IVA, and G6/8A sequences. Samples were annealed in either 1 mM or 100 mM KCl, with or without crowding agents (10% PEG8,000 or 40% PEG200). A fluorescence peak at 610 nm indicates G4 formation. (**B**) CD spectra of dsBcl3 WT, GI/IVA, and G6/8A annealed in 1 mM or 100 mM KCl, with or without 10% PEG8,000, to assess G4 structural signatures. (**C, D)** Atomic force microscopy (AFM) images of dsBcl3 WT, G6/8A, and GI/IVA samples prepared in 1 mM KCl (**C**) or 100 mM KCl (**D**). AFM was performed in air using intermittent tapping mode. (**E**) Quantification of the average maximum height of DNA particles under each condition. Oligos (10 μM) were annealed under the indicated conditions and diluted to 0.5 nM. Samples were deposited on freshly cleaved mica in buffer containing 25 mM KCl and 10 mM MgCl₂. Representative DNA particle images and corresponding height profiles are shown; similar structures are highlighted in boxes. Images were processed using JPKSPM Data Processing Software (v.6.1.158).


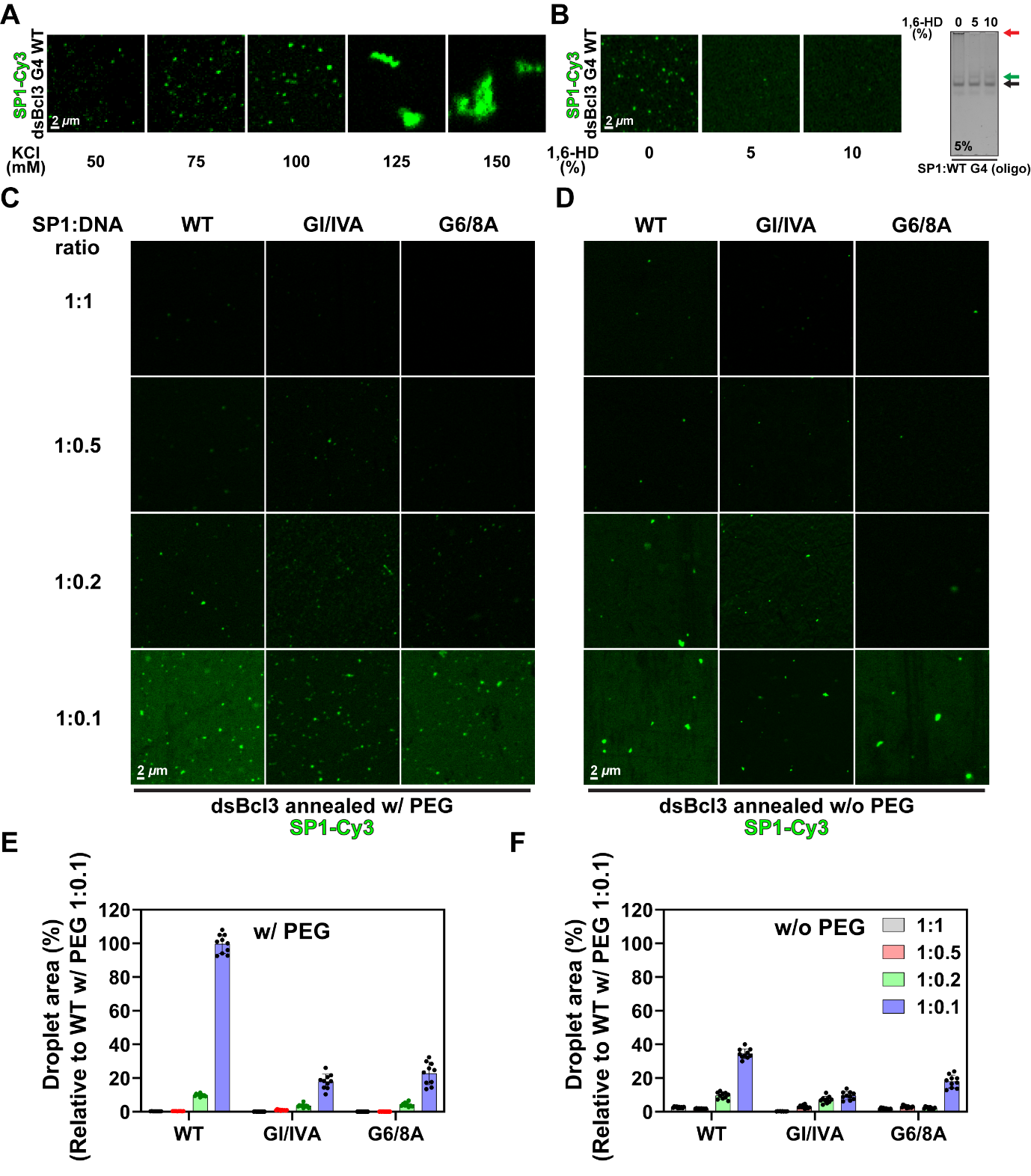


**Supplementary Figure S8. Effects of *Bcl3* double-stranded WT and mutant oligonucleotides on SP1 condensation.**

(**A**) *In vitro* condensation assay of SP1-Cy3 with dsBcl3 WT annealed at varying KCl concentrations to evaluate salt-dependent phase separation. (**B**) **Left:** *In vitro* condensation assay of SP1-Cy3 with dsBcl3 WT in the presence of increasing concentrations of 1,6-hexanediol. **Right:** EMSA of SP1 binding under the same conditions, analyzed on a 5% native polyacrylamide gel. Red, green, and black arrows indicate SP1:G4 condensates, SP1:G4 complexes, and G4 alone, respectively **(C, D)** *In vitro* condensation assay of SP1-Cy3 with dsBcl3 WT, GI/IVA, and G6/8A mutants, annealed in the presence (**C**) or absence (**D**) of 10% PEG8,000. (**E, F**) Quantification of SP1-Cy3 droplet area under the conditions shown in (C) and (D), respectively. Total condensate area is normalized to that observed with dsBcl3 WT at an SP1:DNA ratio of 1:0.1 (with PEG). Data represent mean ± standard deviation from ten randomly selected regions. Experiments were independently performed twice.


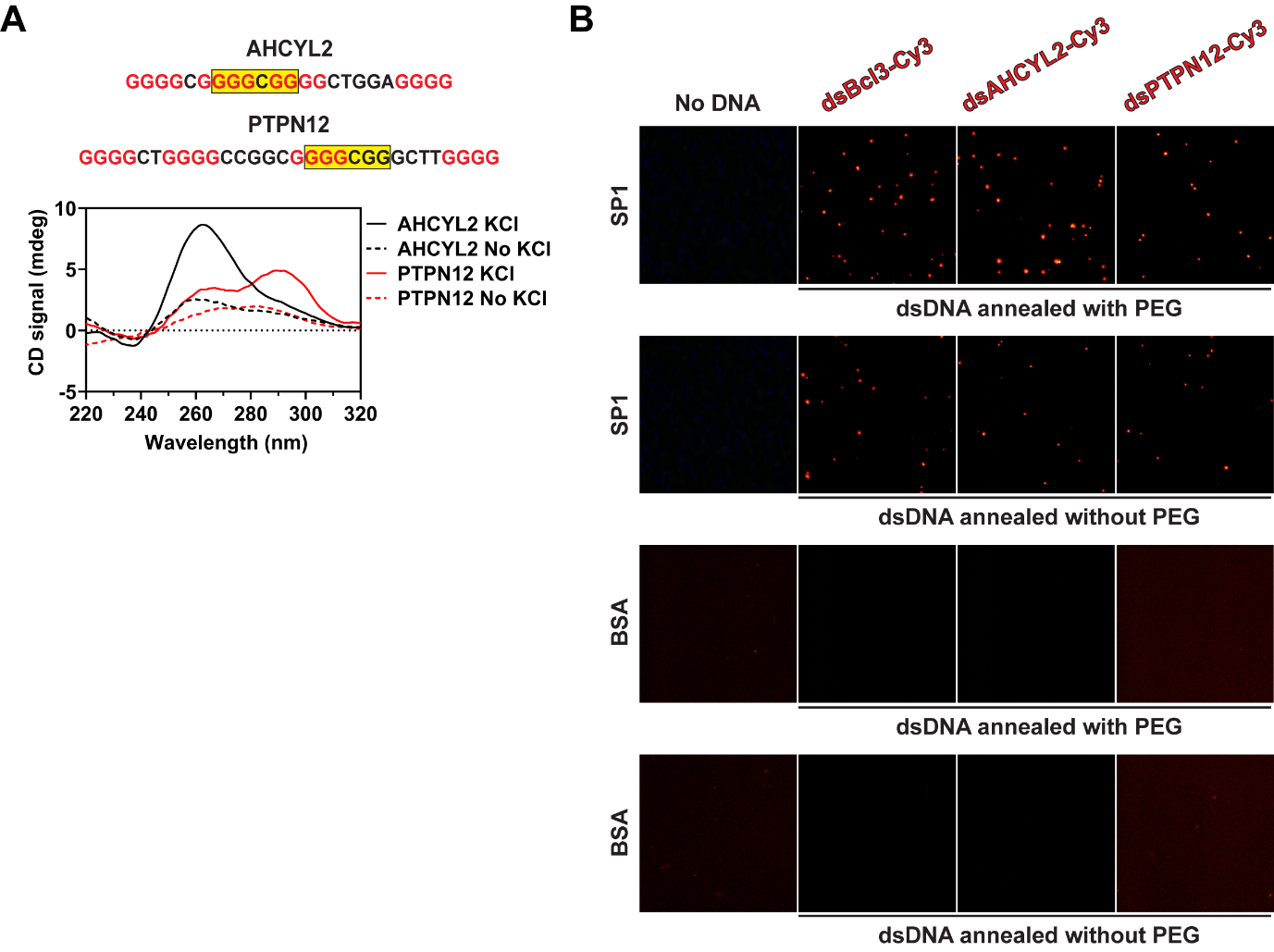


**Supplementary Figure S9. SP1 condensation with *Ahcyl2* and *Ptpn12* promoters harboring G4-forming sequences.** (**A**) CD spectra of oligonucleotides from the *Ahcyl2* and *Ptpn12* promoter regions, annealed in the presence (solid line) or absence (dashed line) of 100 mM KCl. The G4-forming sequences are shown above the plots, with SP1 consensus binding motifs highlighted. (**B**) Representative fluorescence microscopy images of in vitro condensation assays using SP1 and Cy3-labeled double-stranded promoter DNAs (83 bp) from *Bcl3*, *Ahcyl2*, and *Ptpn12*. All DNAs were annealed in 1 mM KCl with 10% PEG8,000. SP1 and DNA were incubated at a 1:0.1 molar ratio. BSA was included as a negative control, showing no condensate formation with DNA.


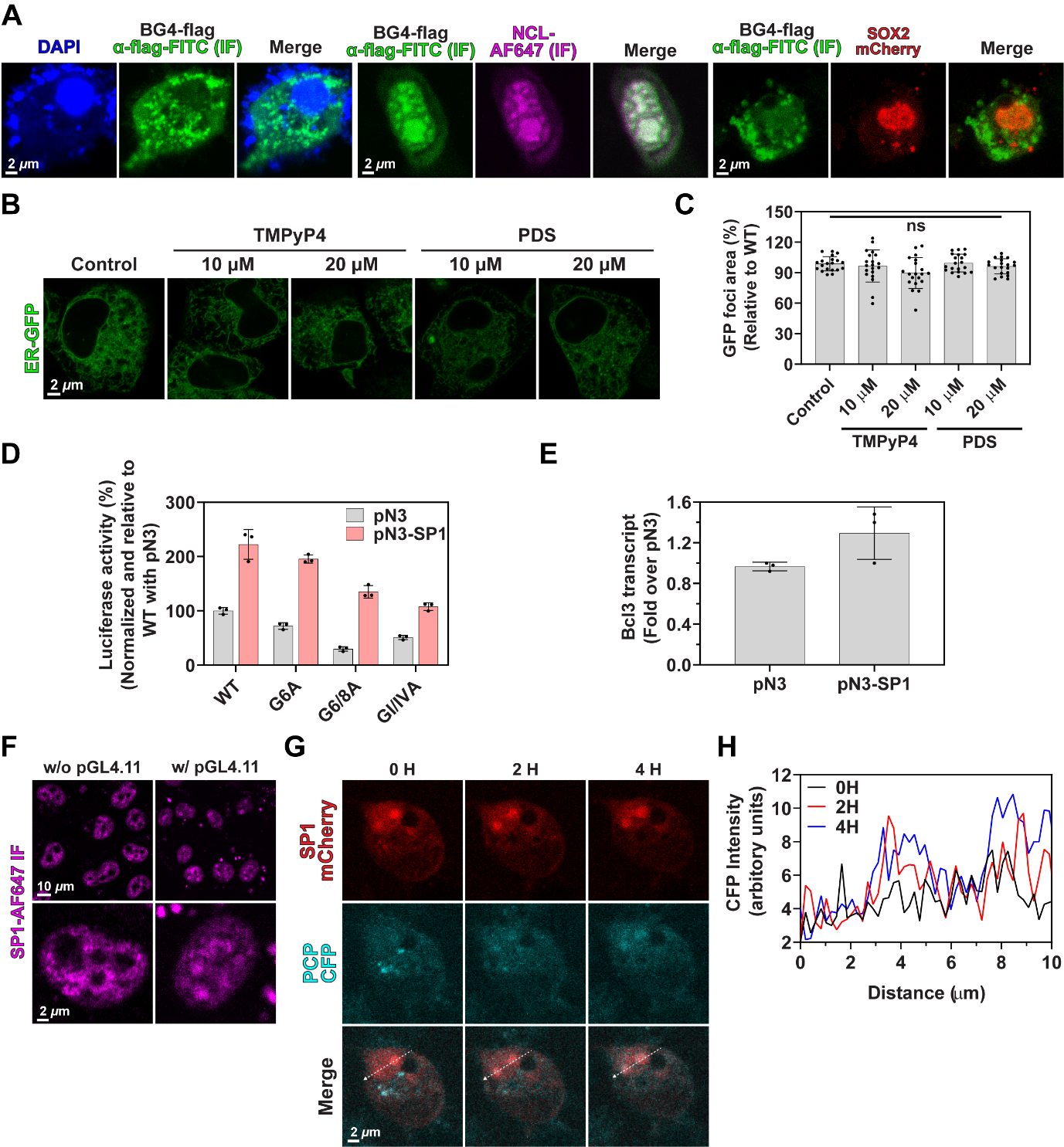


**Supplementary Figure S10. SP1 condensation facilitates transcriptional activation.** (**A**) **Left:** Representative IF images of MDA-MB-231 cells stained with FITC-labeled BG4 antibody to detect G4 structures. **Middle:** Co-staining with Alexa Fluor 647-labeled Nucleolin (AF647-NCL) as a positive control for G4 recognition. **Right:** Overexpression of mCherry-SOX2 as a negative control. Nuclei were stained with DAPI. Images were captured at 40× magnification; close-up views of individual cells are shown. **(B)** IF images of MDA-MB-231 cells expressing GFP with an N-terminal ER signal sequence in the presence of 10 or 20 μM TMPyP4 or PDS. **(C)** Quantification of GFP foci area from (B), shown relative to the untreated control. Data represent mean ± standard deviation. **(D)** Luciferase reporter assay using *Bcl3* WT or MT promoter constructs (pGL4.11) co-transfected with pN3-SP1 or empty pN3 vector in MDA-MB-231 cells. Luciferase activity was normalized to Renilla luciferase from co-transfected pRL-TK plasmid and expressed relative to WT+pN3 control. Graphs represent mean ± standard deviation from at least three independent experiments. **(E)** Quantitative PCR (qPCR) analysis of *Bcl3* transcript levels in MDA-MB-231 cells overexpressing SP1. Transcript levels were normalized to *β-actin*, and fold changes were calculated using the *ΔΔ*Ct method. **(F)** IF images of MDA-MB-231 cells stained with anti-SP1-Alexa Fluor 647 antibody, transfected either with (w pGL4.11) or without (w/o pGL4.11) the pGL4.11-WT-12×PP7 reporter plasmid. Top: 40× magnification; bottom: close-up images of single cells. **(G)** Time-lapse confocal imaging of MDA-MB-231 cells transfected with SP1-mCherry, PCP-CFP, and the pGL4.11-WT-12×PP7 reporter plasmid. SP1-mCherry and the reporter were transfected 24 h prior to PCP-CFP plasmid transfection. Cells were imaged every 2 hours over a 6-hour period at 37 °C with 5% CO₂. Representative 40× magnification and zoomed-in images are shown. **(H)** Linear fluorescence intensity profiles of PCP-CFP along the indicated line in (G), shown at 0, 2, and 4 hours post-transfection.

**
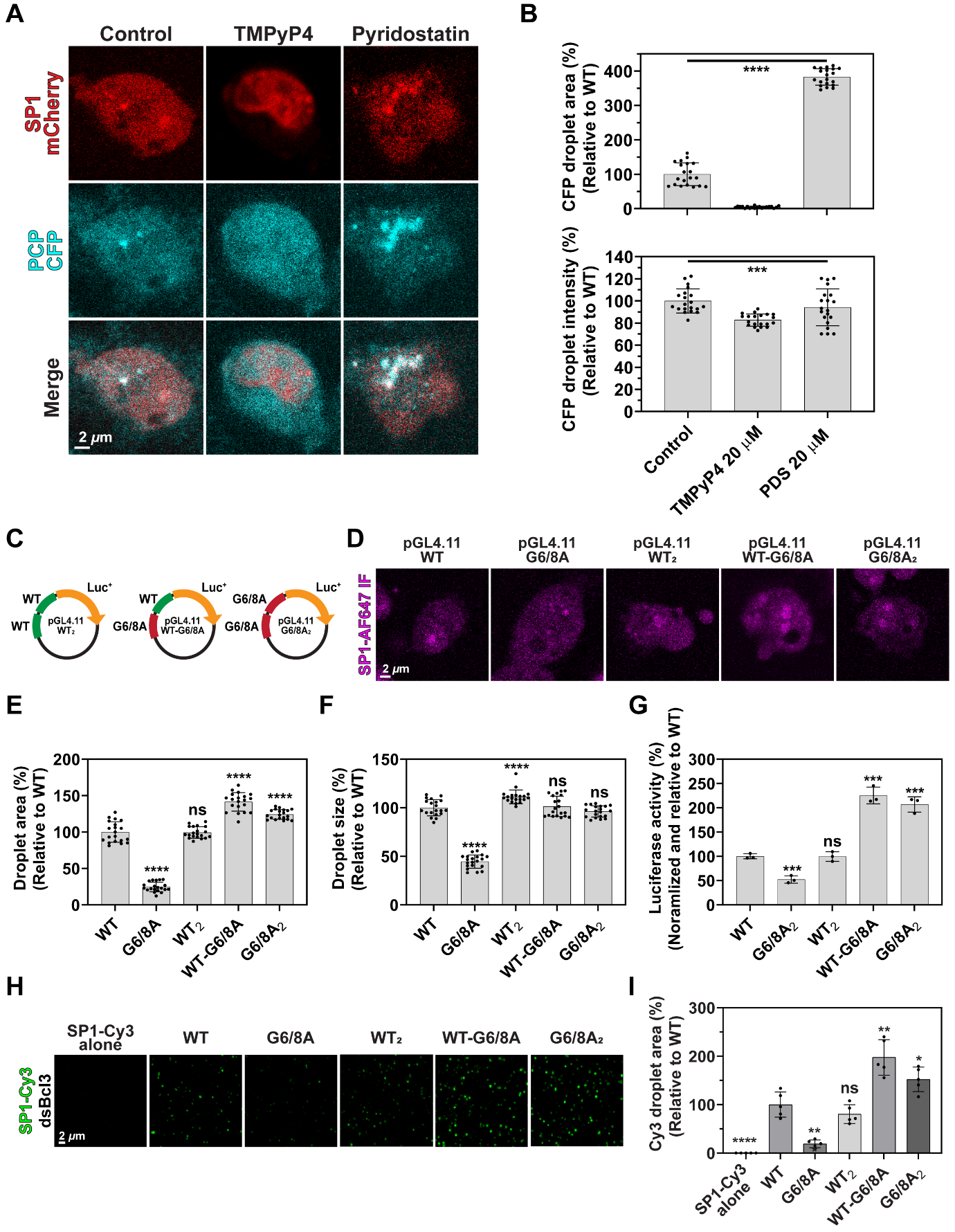
**

**Supplementary Figure S11. Modulation of SP1-mediated transcription by G4 ligands and synthetic combinations of *Bcl3* promoters. (A)** Representative FS images of MDA-MB-231 cells co-transfected with SP1-mCherry and PCP-CFP and treated with 20 μM TMPyP4 or PDS. Images were captured at 40× magnification, with close-up views of individual cells. **(B)** Quantification of PCP-CFP condensate area (top) and droplet intensity (bottom) relative to untreated controls. Graphs show mean ± standard deviation from ten randomly selected regions. Experiments were independently performed twice. **(C)** Schematic representation of pGL4.11 reporter plasmids containing combinations of *Bcl3* promoter sequences: WT-WT (WT²), WT–G6/8A, and G6/8A–G6/8A (G6/8A²). **(D)** IF images of MDA-MB-231 cells stained with anti-SP1-Alexa Fluor 647 antibody (magenta) following transfection with pGL4.11-WT, -WT², -WT–G6/8A, or -G6/8A² reporter plasmids. Images were acquired at 40× magnification, with close-up views of single cells. **(E, F)** Quantification of SP1 condensate total area (E) and average droplet size (F), shown relative to cells transfected with pGL4.11-WT. Graphs display mean ± standard deviation from ten randomly selected regions. Experiments were independently repeated twice. **(G)** Luciferase reporter assay using pGL4.11-WT, -WT², -WT–G6/8A, or -G6/8A² constructs in MDA-MB-231 cells. Luciferase activity was normalized to Renilla luciferase from co-transfected pRL-TK plasmid and presented relative to pGL4.11-WT. Data represent mean ± standard deviation from at least three independent experiments. **(H)** *In vitro* condensation assay of SP1-Cy3 with dsDNA templates (WT, G6/8A, WT², WT–G6/8A, G6/8A²) annealed in 10% PEG8,000 with 1 mM KCl. **(I)** Quantification of total SP1-Cy3 condensate area relative to WT. Graphs show mean ± standard deviation from five randomly selected regions.


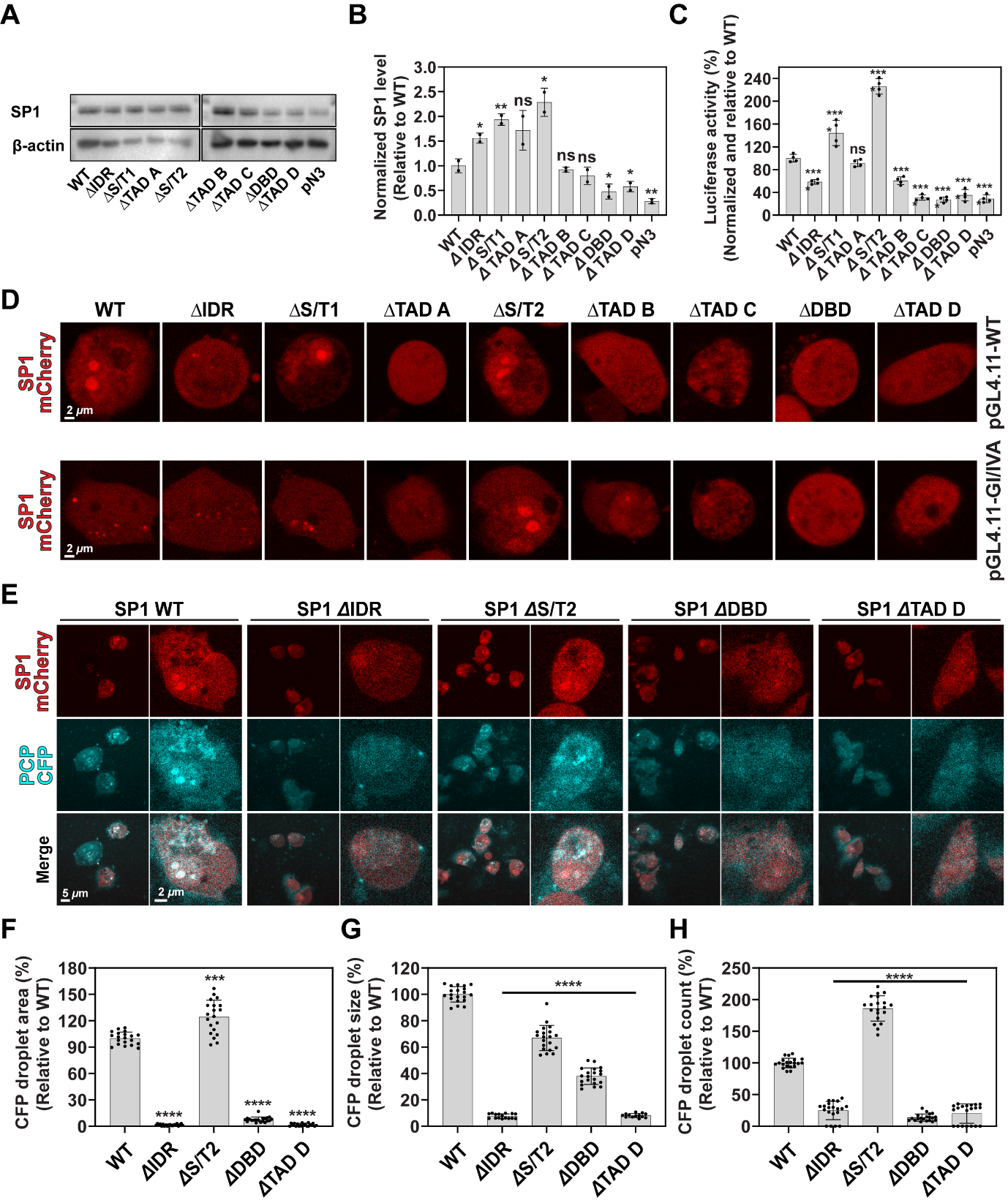


**Supplementary Figure S12. The N-terminal intrinsically disordered region (IDR), transactivation domains (TADs), and DNA-binding domain (DBD) of SP1 are essential for SP1 condensation-dependent transcription. (A)** Western blot analysis of lysates from MDA-MB-231 cells transfected with pN3 constructs expressing SP1 WT or deletion mutants: ΔIDR, ΔTAD A, ΔS/T1, ΔTAD B, ΔS/T2, ΔTAD C, ΔDBD, or ΔTAD D. **(B)** Quantification of SP1 protein levels normalized to β-actin, shown relative to SP1 WT. **(C)** Luciferase reporter assay using the pGL4.11-*Bcl3* WT promoter in MDA-MB-231 cells co-transfected with pN3-SP1 WT or the indicated SP1 deletion mutants. Luciferase activity was normalized to Renilla luciferase from co-transfected pRL-TK and shown relative to SP1 WT. Graphs represent mean ± standard deviation from at least three independent experiments. **(D)** Representative FS images of MDA-MB-231 cells transfected with mCherry-tagged SP1 WT or mutant constructs and either the pGL4.11-*Bcl3* WT or GI/IVA reporter plasmid. **(E)** FS images of MDA-MB-231 cells co-transfected with PCP-CFP and mCherry-tagged SP1 WT, ΔIDR, ΔS/T2, ΔDBD, or ΔTAD D constructs. Left panels: 40× magnification; right panels: enlarged images of individual cells. **(F–H)** Quantification of PCP-CFP condensate properties from (E): **(F)** total condensate area, **(G)** average droplet size, and **(H)** droplet count, each shown relative to WT SP1-mCherry. Graphs display mean ± standard deviation from ten randomly selected regions. Experiments were independently performed twice.


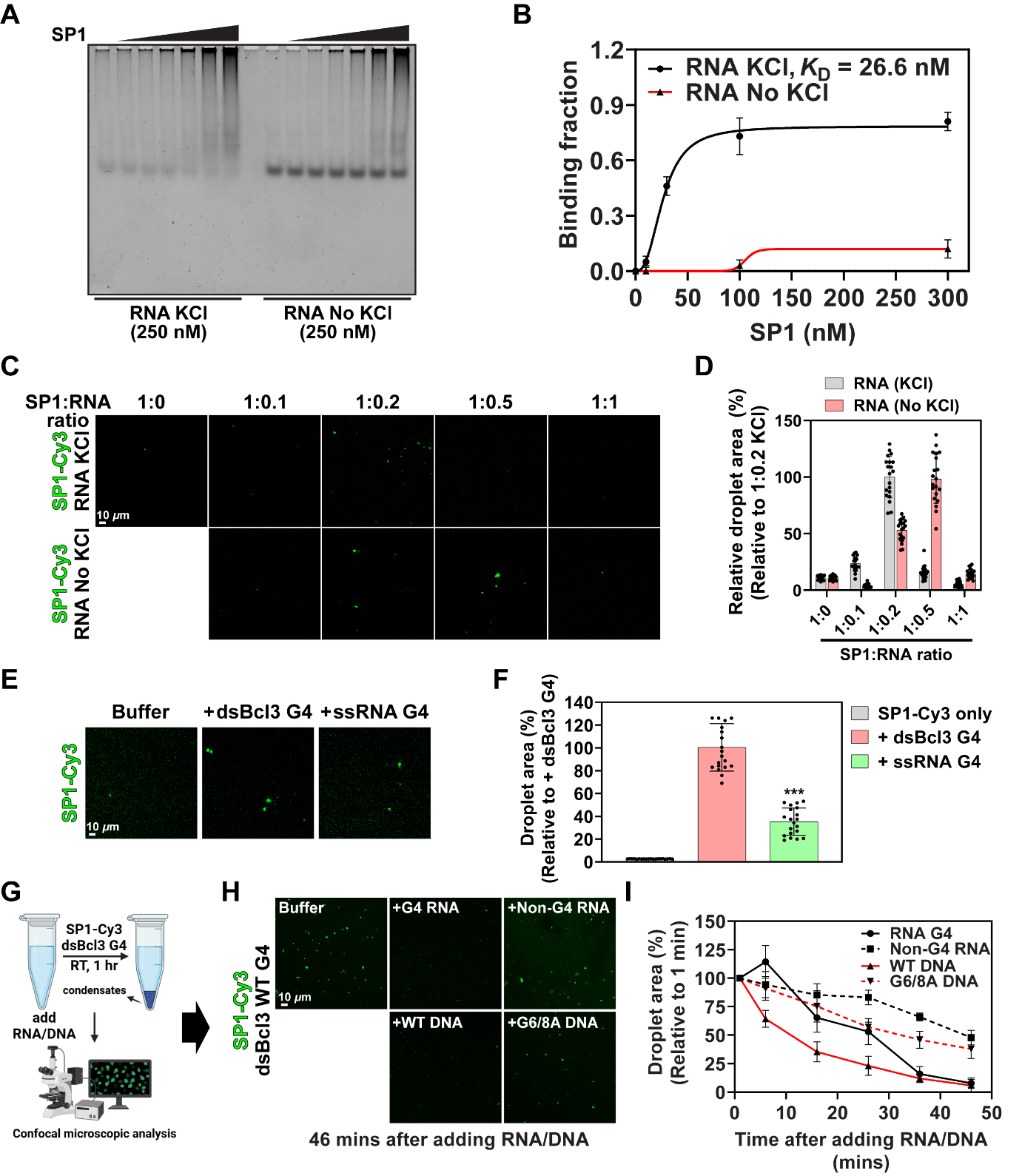


**Supplementary Figure S13. Effects of excess RNA and DNA on SP1:G4 condensates.** (**A**) EMSA of G4 RNA and non-G4 RNA oligos incubated with increasing concentrations of SP1. Samples were resolved on a 5% native polyacrylamide gel. Bands migrating more slowly than free RNA indicate SP1-bound complexes. (**B**) Quantification of RNA binding fractions calculated as: 1 – (free RNA intensity / free RNA intensity at 0 nM SP1). Apparent dissociation constants (*K*_D_) were determined by fitting binding curves to the Hill equation using nonlinear regression in GraphPad Prism. Graphs represent mean ± standard deviation from at least two independent experiments. (**C**) Representative FS images of SP1-Cy3 condensates formed at different SP1:G4 RNA ratios. G4 RNA was annealed in the presence (RNA KCl) or absence (RNA No KCl) of 100 mM KCl. (**D**) Total condensate area is shown relative to that at an SP1:G4 RNA ratio of 1:0.2 (with KCl). Graphs display mean ± standard deviation from ten randomly selected regions. Experiments were independently performed twice. (**E**) Representative FS images of SP1-Cy3 condensates formed with either double-stranded *Bcl3* G4 DNA (dsBcl3 G4) or single-stranded G4 RNA (ssRNA G4). (**F**) Quantification of total condensate area relative to SP1:dsBcl3 G4 condition. Data represent mean ± standard deviation from ten randomly selected regions. Experiments were independently performed twice. (**G**) Schematic of experimental design for testing SP1 condensate dissolution by DNA or RNA. (**H**) Representative FS images of SP1-Cy3 condensates 45 minutes after the addition of dsBcl3 WT, G6/8A, G4 RNA, or non-G4 RNA. (**I**) Time-lapse analysis of SP1-Cy3 condensate area following the addition of DNA or RNA at 0 minutes. Dashed lines represent non-G4 controls (dsBcl3 G6/8A and non-G4 RNA). Graphs show mean ± standard deviation from five randomly selected regions.


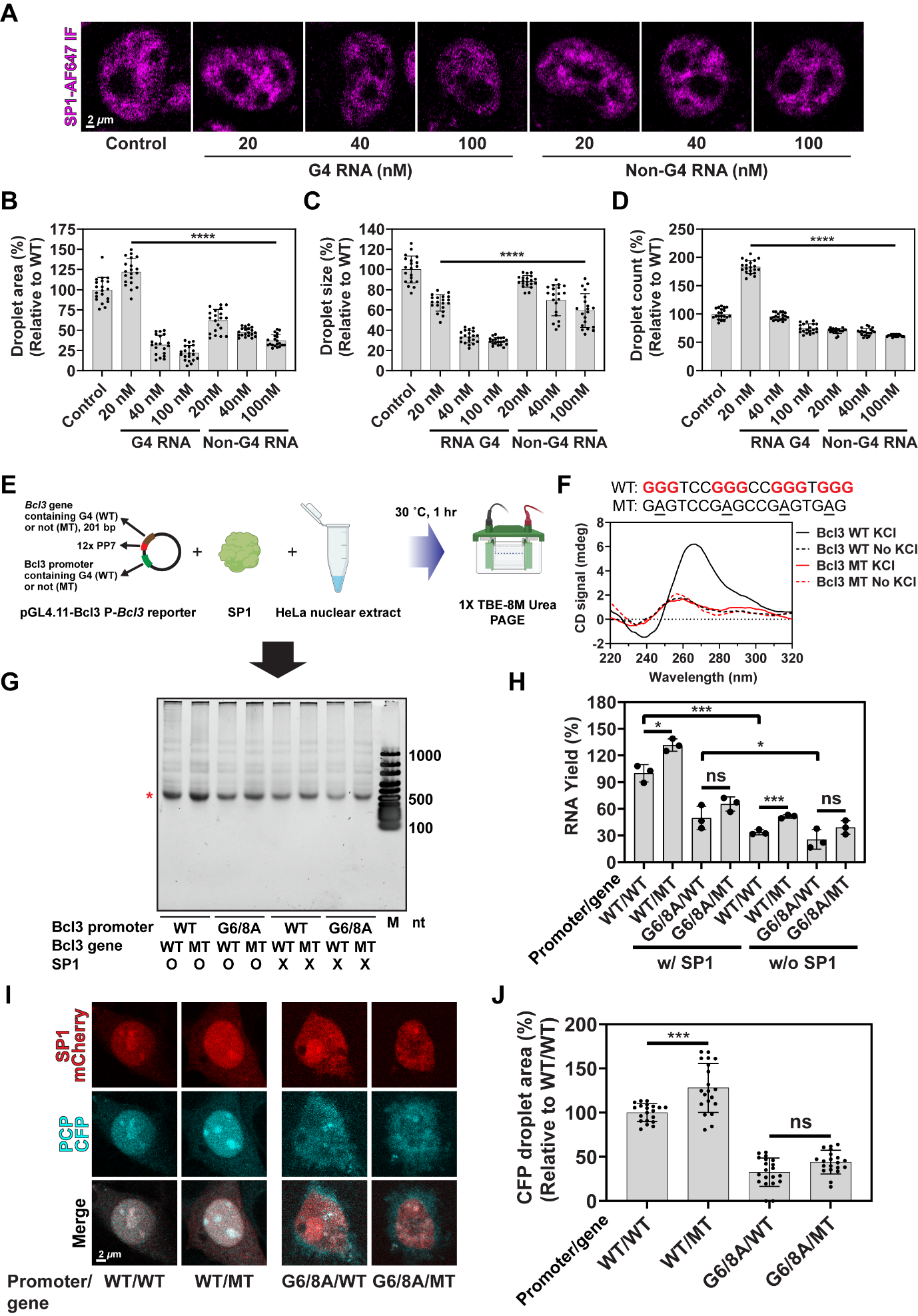


**Supplementary Figure S14. Effects of excess RNA and DNA on SP1:G4 condensates and transcriptional output. (A)** IF images of MDA-MB-231 cells stained with anti-SP1-Alexa Fluor 647 antibody (magenta) following treatment with increasing concentrations of G4 RNA or non-G4 RNA. Images were captured at 40× magnification; close-up views of single cells are shown. **(B–D)** Quantification of SP1 condensates from (A), showing **(B)** total condensate area, **(C)** droplet size, and **(D)** droplet count, each normalized to untreated control. Graphs represent mean ± standard deviation from ten randomly selected regions. Experiments were independently performed twice. **(E)** Schematic of the in vitro transcription assay using HeLa nuclear extract and *Bcl3* reporter constructs. **(F)** CD spectra of the G4-forming sequence from the *Bcl3* gene and its mutant, confirming G4 structure. **(G)** Urea-PAGE analysis of *in vitro* transcription using pGL4.11-*Bcl3*P-*Bcl3* gene reporter plasmids. A transcript of ~500 nt—corresponding to the 12×PP7 cassette (312 nt) and the G4-containing region (201 nt)—is marked with a red asterisk. **(H)** Quantification of RNA yield from (G), calculated from band intensities and shown relative to the WT/WT construct in the presence of SP1. **(I)** Representative FS images of MDA-MB-231 cells co-transfected with mCherry-SP1, PCP-CFP, and *Bcl3*P-*Bcl3* gene reporter plasmids containing WT or mutant G4 sequences. **(J)** Quantification of PCP-CFP condensate area from (I), normalized to the WT/WT reporter condition. Graphs represent mean ± standard deviation from ten randomly selected regions. Experiments were independently performed twice.

**Supplementary Table S1. Oligos used in this study**.

| **Oligos** | **Sequences (5` to 3`)** |
| --- | --- |
| **Oligos for ssDNA G4s** | |
| WT | CGGGGGGGCCGGGGGCGGGGAGGCGGGCGG |
| G6A | CGGGGGGGCCGGAGGCGGGGAGGCGGGCGG |
| G6/8A | CGGGGGGGCCGGAGGCGGAGAGGCGGGCGG |
| GI/IVA | CGGAAAGGCCGGGGGCGGGGAGGCAAACGG |
| PC_NG4 | CGGAGAGACCGGGGGCGGGGAGACGAGCAG |
| Negative control (NC) | CGGAGAGACCGAGAGCAGAGAGACGAGCAG |
| **Oligos for dsDNA G4s** | |
| dsBcl3_WT forward | CAGGAAACCCCTGGGGCGTACGGGTGGCCCC  GGGGGGGCCGGGGGCGGGGAGGCGGGCGG  CCGGCACCGCCCCGGCCGACAAAAGTCCCT |
| dsBcl3_WT reverse | AGGGACTTTTGTCGGCCGGGGCGGTGCCGGC  CGCCCGCCTCCCCGCCCCCGGCCCCCCCGGG  GCCACCCGTACGCCCCAGGGGTTTCCTG |
| dsBcl3_G6/8A forward | CAGGAAACCCCTGGGGCGTACGGGTGGCCC  CGGGGGGGCCGGAGGCGGAGAGGCGGGCGG  CCGGCACCGCCCCGGCCGACAAAAGTCCCT |
| dsBcl3_G6/8A reverse | AGGGACTTTTGTCGGCCGGGGCGGTGCCGGC  CGCCCGCCTCTCCGCCTCCGGCCCCCCCGGG  GCCACCCGTACGCCCCAGGGGTTTCCTG |
| dsBcl3_GI/IVA forward | CAGGAAACCCCTGGGGCGTACGGGTGGCCCC  GGAAAGGCCGGGGGCGGGGAGGCAAACGGCC  GGCACCGCCCCGGCCGACAAAAGTCCCT |
| dsBcl3_GI/IVA reverse | AGGGACTTTTGTCGGCCGGGGCGGTGCCGGCC  GTTTGCCTCCCCGCCCCCGGCCTTTCCGGGGCC  ACCCGTACGCCCCAGGGGTTTCCTG |
| AHCYL2 forward | GTGGCGCCTCGCGCGAGGCCAGACCGGGACGG  GGCGGGGCGGGGCTGGAGGGGCCGGGACTGG  CGGGCGGAGAAAGGGGGTGG |
| AHCYL2 reverse | CCACCCCCTTTCTCCGCCCGCCAGTCCCGGCCC  CTCCAGCCCCGCCCCGCCCCGTCCCGGTCTGGC  CTCGCGCGAGGCGCCAC |
| PTPN12 forward | CCGCTGAAGTGCCTTCCAGCCACTCCAAGCGGGG  CTGGGGCCGGCGGGGCGGGCTTGGGGGCGTGGC  CGGGAGGCGGGCGGGGATGCATC |
| PTPN12 reverse | GATGCATCCCCGCCCGCCTCCCGGCCACGCCCCC  AAGCCCGCCCCGCCGGCCCCAGCCCCGCTTGGA  GTGGCTGGAAGGCACTTCAGCGG |
| **Oligos for cloning** | |
| SP1 forward | GTGATCGTCTAGAATGAGCGACCAAGATCAC |
| SP1 reverse | GGTTATAGCTAGCGAAGCCATTGCCACTGAT |
| BG4 forward (with Kozak sequence) | AGATATAGCGATCGCGCCACCATGGCCGAGGTGCAG |
| BG4 reverse | GAGCGCGCGTACGGGGCCGCACCTAGGAC |
| **Oligos for site-directed mutagenesis** | |
| ΔIDR forward | GGTATGGACCTCACAGCCACACAA |
| ΔIDR reverse | GAGGTCCATACCGGTGGTGGCTCT |
| ΔS/T1 forward | TGGCAGGTCTCTGGTGGGCAGTAT |
| ΔS/T1 reverse | AGAGACCTGCCAGCCATTGGCACC |
| ΔTAD A forward | GGTGGGTATGTGACCAATGTACCA |
| ΔTAD A reverse | CACATACCCACCAGAGACTGTGCG |
| Continued. |  |
| **Supplementary Table 1. Continued.** |  |
| ΔS/T2 forward | TACTCACAGCAACAAATTCTTATC |
| ΔS/T2 reverse | TTGCTGTGAGTAGCTATTGGCATT |
| ΔTAD B forward | CAGACAAGCAGCAGCAACACCACT |
| ΔTAD B reverse | GCTGCTTGTCTGCTGGTTTTGCTC |
| ΔTAD C forward | CAGACCGACAGTGAAGGAAGGGGC |
| ΔTAD C reverse | ACTGTCGGTCTGCCCCAAGGAAAC |
| ΔIDR forward | CCGTTCATCGACGCGGCCAAGCGGCTG |
| ΔIDR reverse | CAGCCGCTTGGCCGCGTCGATGAACGG |
| ΔDBD forward | AAACAGCAGAATAAGAAGGGAGGC |
| ΔDBD reverse | ATTCTGCTGTTTCTTTTTGCCAGG |
| ΔTAD D forward | TCAAGACCTTGGCTAGCGAGGGCAGAGTGGA |
| ΔTAD D reverse | CTAGCCAAGGTCTTGATATGTTTTGACAGGTGGTC |
| **ssRNA oligos** | |
| G4 RNA | GGGTCCGGGCCGGGTGGG |
| Non-G4 RNA | AAATCCAAACCAAATAAA |
| **Oligos for qRT-PCR** | |
| Bcl3 forward | CCTATACCCCATGATGTGCC |
| Bcl3 reverse | GCACCACAGCAATATGGAGA |
| β-actin forward | CACCATTGGCAATGAGCGGTTC |
| β-actin reverse | AGGTCTTTGCGGATGTCCACGT |
| Bcl3 promoter forward | GGCCGACAAAAGTCCCTTCA |
| Bcl3 promoter reverse | GGGAGGGTGGTTTCGCC |
| cMyc promoter forward | GATTTCTCCCAAACCCGGCA |
| cMyc promoter reverse | CTGCTCAGGCTTCCGTGG |
| ESR1 promoter forward | AGAAAATCGGCTGGATGGCA |
| ESR1 promoter reverse | TCGGTAGACAGGAGGCTGTT |
| SP1 promoter forward | CCTTCCAAGCCAATCATCTCC |
| SP1 promoter reverse | CGCCACAAGCCCAACCTA |
| RINL promoter forward | AGCCGACTAGCCTATCTCCTT |
| RINL promoter reverse | CAGAGAGGCTCCTGACGTTG |
